# Unexpected Resistance to Base-Catalyzed Hydrolysis of Nitrogen Pyramidal Amides Based on the 7-Azabicyclic[2.2.1]heptane Scaffold

**DOI:** 10.3390/molecules23092363

**Published:** 2018-09-15

**Authors:** Diego Antonio Ocampo Gutiérrez de Velasco, Aoze Su, Luhan Zhai, Satowa Kinoshita, Yuko Otani, Tomohiko Ohwada

**Affiliations:** 1 Laboratory of Organic and Medicinal Chemistry, Graduate School of Pharmaceutical Sciences, University of Tokyo, 7-3-1 Hongo, Bunkyo-ku, Tokyo 113-0033, Japan; daogv1@hotmail.com (D.A.O.G.d.V.); aaronsusu@gmail.com (A.S.); zhailuhanzlh@gmail.com (L.Z.); s.kinoshita13@gmail.com (S.K.); otani@mol.f.u-tokyo.ac.jp (Y.O.); 2 Department of Chemistry, St John’s College, University of Cambridge, St John’s Street, Cambridge CB2 1TP, UK

**Keywords:** non planar amide, base-catalyed hydrolysis, water solvation, entropy

## Abstract

Non-planar amides are usually transitional structures, that are involved in amide bond rotation and inversion of the nitrogen atom, but some ground-minimum non-planar amides have been reported. Non-planar amides are generally sensitive to water or other nucleophiles, so that the amide bond is readily cleaved. In this article, we examine the reactivity profile of the base-catalyzed hydrolysis of 7-azabicyclo[2.2.1]heptane amides, which show pyramidalization of the amide nitrogen atom, and we compare the kinetics of the base-catalyzed hydrolysis of the benzamides of 7-azabicyclo[2.2.1]heptane and related monocyclic compounds. Unexpectedly, non-planar amides based on the 7-azabicyclo[2.2.1]heptane scaffold were found to be resistant to base-catalyzed hydrolysis. The calculated Gibbs free energies were consistent with this experimental finding. The contribution of thermal corrections (entropy term, *–TΔS***^‡^**) was large; the entropy term (*ΔS***^‡^**) took a large negative value, indicating significant order in the transition structure, which includes solvating water molecules.

## 1. Introduction

**Scheme 1:**
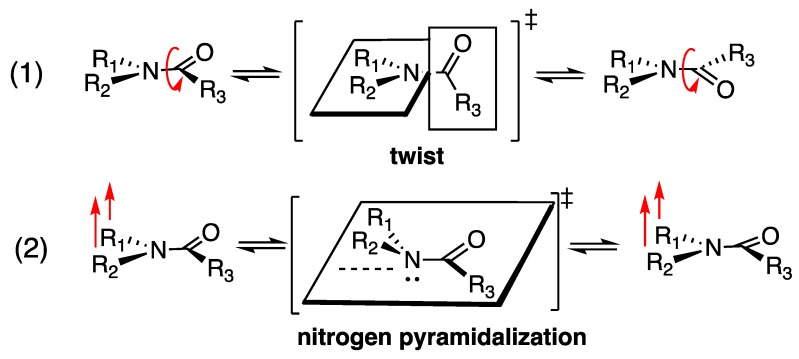
Amide transformation processes causing non-planarity: (**1**) N-C bond twisting (rotation) and (**2**) nitrogen pyramidalization. These transformations are interconnected.

In non-planar amides, distortion of the amide bond can arise from both twisting about the C-N bond and pyramidalization at the nitrogen atom (Scheme 1) [1,2]. These transformations of the amide bond are essentially mutually correlated, and the transition states of the amide rotation involved bond twisting and nitrogen pyramidalization at the same time [3] (see also Reference [4]). The partial double-bond character of planar amides limits rotation about the C-N bond, and this feature also contributes to stabilization, due to electron-delocalization. Decrease in *sp^2^* nitrogen character, with increase of *sp^3^* character, tends to weaken the C-N bond and increase the electrophilicity of the carbonyl carbon atom [1].

**Figure 1:**
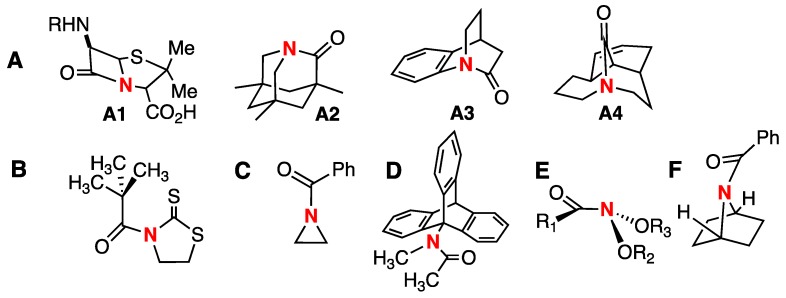
Some examples of non-planar amides.

Non-planar amides are usually transitional structures that are involved in amide bond rotation (Scheme 1 (**1**)) and inversion of the nitrogen atom (Scheme 1 (**2**)). However, even in ground-minimum structures, amide distortion can be caused by several different factors, as illustrated in Figure 1A lactam ring strain of the nitrogen atom at a bridgehead position [5,6,7,8]; Figure 1B steric repulsion between substituents at the carbonyl and nitrogen positions [9]; Figure 1C angle strain at the nitrogen position [10]; Figure 1D bulkiness of substituents at the nitrogen position [11]; Figure 1E anomeric effect [12]; Figure 1F 1,3-allylic strain with respect to the pseudo C-N double bond [4,13]. These compounds are examples of ground-minimum non-planar amides. One of the most significant consequences of losing planarity of amides is an increase in lability: Reduction of the amide resonance exposes the carbonyl functionality to nucleophilic attack and acyl transfer reaction. In particular, hydrolysis by water under both acidic and basic conditions, and even under neutral conditions, is greatly accelerated when planarity is disrupted [5,6,7,8,9].

**Figure 2:**
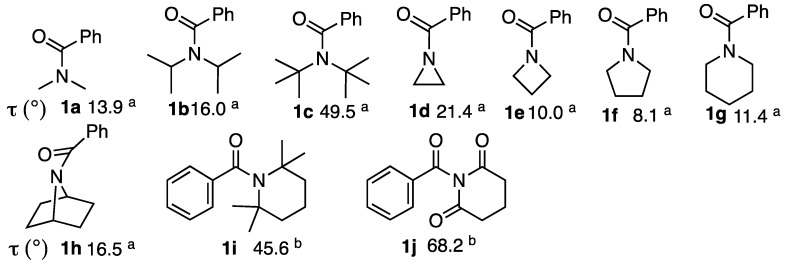
Distortion angles (τ) of ground-state-stable benzoyl amides. ^a^ B3LYP/6-31 + G(d) level of theory (Reference [14]). ^b^ X-ray data (Reference [15]). Calculated dihedral angle τ. τ = (ω_1_ + ω_2_)/2 (ω_1_ = ∠R-N-C-O and ω_2_ = ∠R-N-C-R’) [14].

The torsional angle (τ) (the mean twisting angle around the C-N bond, see Figure 2) for completely planar amides is 0.0°. It is clear that the τ angle of ground-minimum non-planar amides can adopt values different from zero. Stable ground-state *N*,*N*-disubstituted tertiary amides, such as benzamide derivatives (**1a**–**1j**) can also take non-zero τ values (Figure 2), as their calculated structures show distortions from planarity [14,15]. Some of them (**1c**, **1l** and **1j**) are activated for facile cleavage of the amide C-N bond in the presence of various catalysts [16,17,18,19]. 

**Figure 3:**
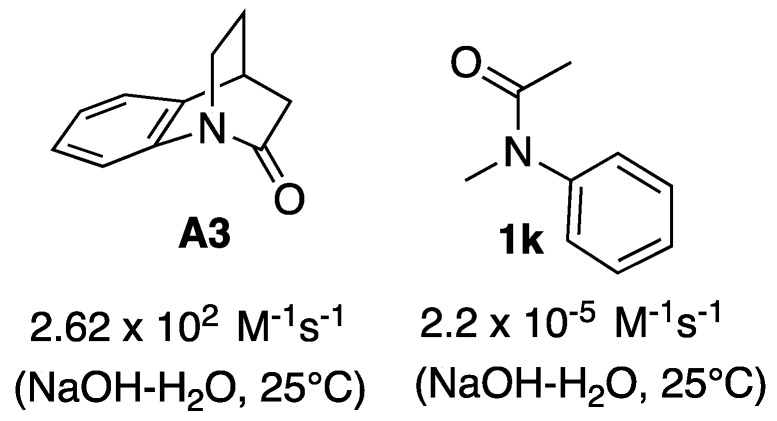
Model molecules used by Brown in his comparative kinetics study.

Brown proposed a close relationship between nitrogen pyramidalization, C-N bond length and kinetic reactivity to hydroxy anion attack [7], based on a comparison of the hydrolysis kinetics of analogous planar (molecule **1k** in Figure 3) and non-planar amides (**A3** in Figure 3). The base-catalyzed hydrolysis reaction at 25 °C showed a striking activation by 7 orders of magnitude in passing from the planar to the non-planar structure (see values in Figure 3).

**Figure 4:**
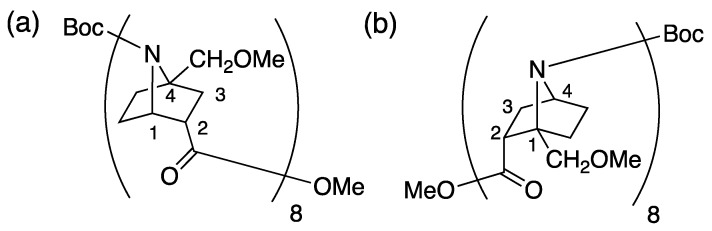
(**a**) Schematic representation of 7-azabicyclo[2.2.1]heptane amide *cis*-(*S*)-8mer. (**b**) Schematic representation of the *trans*-(*R*)-8mer.

Because of the lability of most non-planar amides in the presence of water [5,6,7,8,9], there have been few applications of these scaffolds (Figure 1) in molecular design. On the other hand, while 7-azabicyclo[2.2.1]heptane amides are highly suspicious of chemical stability, due to nitrogen-pyramidalization [12,13], 7-azabicyclo[2.2.1]heptane amides are of interest, beccause they can be regarded as conformationally constrained β-proline mimics. Consequently, several derivatives have been synthesized, and helical structures of homooligomers of β-proline mimics derived from azabicyclo[2.2.1]heptane amide have been reported [20,21]. The helical structures were stable even in the absence of intramolecular hydrogen bonds [22,23]. By introducing suitable bridgehead substituents, either all-*cis* amide or all-*trans* amide conformations were obtained. Conformational control favoring the *cis*-isomer was achieved by introducing substituents at the C-4 bridgehead position (Figure 4(a)). The *cis*-amide structure is heat-stable and the helical structure remains intact in a variety of solvents (water, alcohol, halogenated solvents and cyclohexane) [24]. On the other hand, conformational control favoring the *trans*-isomer was achieved by introducing substituents at the C-1 bridgehead position (Figure 4(b)). The *trans*-amide structure also proved to be heat-stable and the helical structure remained intact in both hydrophilic and hydrophobic solvents [25].

**Figure 5:**
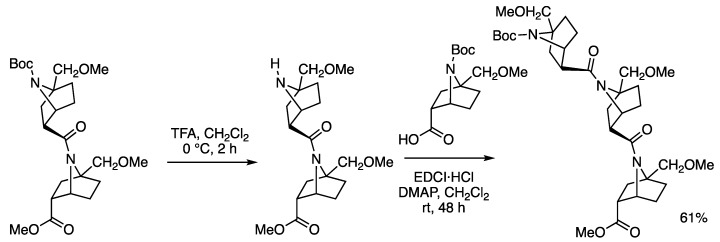
Acidic deprotection procedure in the synthesis of the homooligomers.

In the synthesis of oligomers of 7-azabicyclo[2.2.1]heptane amides (Figure 4) [24,25], acid-catalyzed deprotection of the Boc group was compatible with the amide linkage (Figure 5). Therefore, we thought that the bicyclic amide linkage might be stable under acidic conditions and convectional mild reaction conditions. However, to our knowledge, neither qualitative nor quantitative data about the base-catalyzed hydrolytic reactivity of this system have been reported. 

Therefore, the aim of the present study is to establish the reactivity profile in the base-catalyzed hydrolysis of this 7-azabicyclo[2.2.1]heptane amide system, which might serve as a model for the enzymatic cleavage of peptide bonds. To this end, kinetic studies of the base-catalyzed hydrolysis of the amide of 7-azabicyclo[2.2.1]heptane benzamides were conducted and the results were compared with reported data for related monocyclic amide compounds. Theoretical calculations were also carried out to aid in understanding the unexpectedly low reactivity.

## 2. Results and Discussion

**Figure 6:**
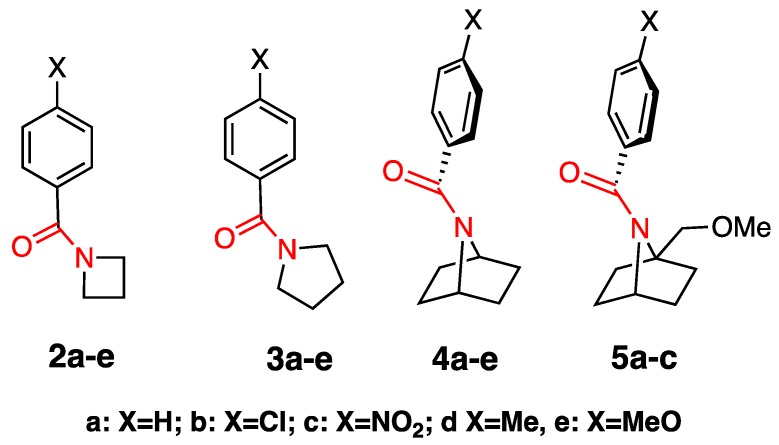
Model molecules used in this study. *N*-Benzoylazetidines **(2a**–**e)** and *N*-benzoylpyrrolidines **(3a**–**e)** were examined as monocyclic amides. *N*-Benzoyl-7-azabicyclo[2.2.1]heptanes (**4a**–**e**) and *N*-benzoyl-1-(methoxymethyl)-7-azabicyclo- [2.2.1]heptanes (**5a**–**c**) were examined as bicyclic amides.

In order to estimate the strength of amide bonding, we compared the reactivities of planar and non-planar amides, specifically pyrrolidine amides (**3a**–**e**) and 7-azabicyclo[2.2.1]heptane amides (**4a**–**e**) (Figure 6). We also evaluated the effect of introducing substituents on the bridgehead position of the bicycle (**5a**–**e**). We utilized azetidine amides (**2a**–**e**) (Figure 6) as reference compounds for non-planar cyclic amides. In general, we found that the base-catalyzed hydrolysis of the bicyclic amides (**4** and **5**) was rather slow. Among aromatic substituents, we focused on **a** (H), **b** (Cl) and **c** (NO_2_) (Figure 6), for which the reaction proceeds at acceptable speed, since the reactions in the cases of substituents **d** (Me) and **e** (MeO) are too slow to obtain kinetic data by means of NMR (see below).

### 2.1. Synthesis 

**Scheme 2:**
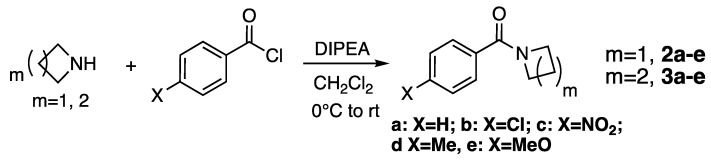
Synthesis of monocyclic amides **2** and **3.**

The monocyclic *N*-benzoylazetidines **2a**–**e** and *N*-benzoylpyrrolidines **3a**–**e** were synthesized in a straightforward manner by coupling the corresponding amines with different benzoyl chlorides (Scheme 2). For the synthesis of the azetidine compounds, the chloride salt of the amine was used as the starting material, with 3 equivalents of DIPEA (diispropylethylamine). For the synthesis of pyrrolidine compounds, 1.2 equivalents of DIPEA sufficed. Compounds were obtained in good yields.

**Scheme 3:**
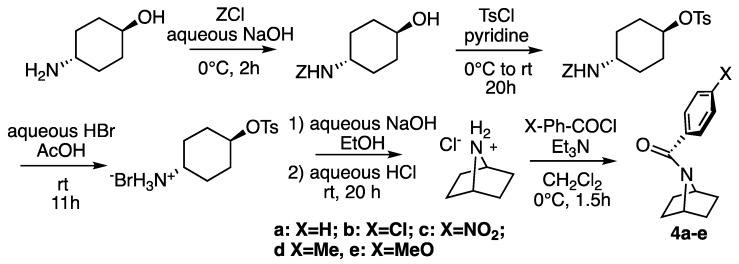
Synthesis of bridgehead-unsubstituted bicyclic amides **4**.

In addition to the monocyclic amides, we synthesized unsubstituted (**4a**–**e**) and substituted (**5a**–**e**) bicyclic amides. *N*-Benzoyl-7-azabicyclo[2.2.1]heptanes were synthesized starting from *trans*-4-aminocyclohexanol (Scheme 3) [4]. The primary amine was substituted by benzyloxycarbonyl chloride (ZCl), and the hydroxy group was changed to toluenesulfonate in order to facilitate bicycle formation. Coupling of benzoyl chloride or *para*-substituted benzoyl chlorides gave the bicyclic amides (**4a**–**e**). 

**Scheme 4:**
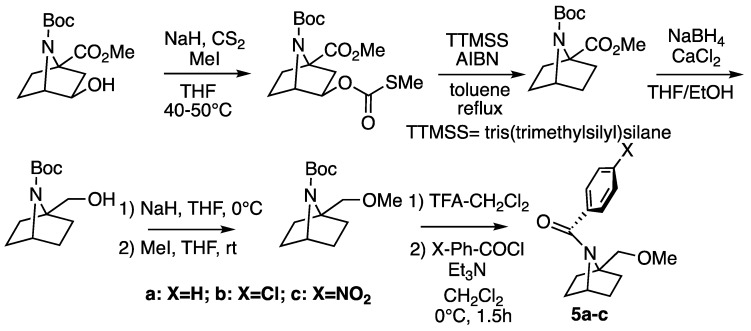
Synthesis of bridgehead-substituted bicyclic amides **5**.

A different strategy was followed for the bridgehead-substituted bicyclic amides **5a**–**c** (Scheme 4). The hydroxy group was removed from the previously synthesized monomer scaffold by Barton–McCombie deoxygenation using AIBN and tris(trimethylsilyl)silane (TTMSS) [26]. After that, the bridgehead ester functionality was first reduced to alcohol and then changed to ether. After Boc-deprotection, the compounds were coupled with various *para*-substituted benzoyl chlorides to afford the bridgehead-substituted bicyclic amides. 

### 2.2. Alkaline Hydrolysis of Planar Amide 3a

In order to assess the chemical reactivities of the non-planar 7-azabicyclo[2.2.1]heptane amides **4** and **5** in alkaline conditions, we first examined the kinetics of the planar amide N-benzoyl pyrrolidine **3a** (X=H) in order to optimize the reaction conditions, because the hydrolysis of **3a** is expected to be the slowest among these compounds (**2**, **3**, **4** and **5** in Figure 5). 

#### 2.2.1. Optimization of Reaction Conditions

Given that hydrolysis involves working with water as a solvent, it was necessary to confirm the solubility of the reactants. In order to carry out the reactions, water-miscible co-solvents had to be chosen. In addition, since alkaline conditions entail high concentrations of hydroxide, some solvents (such as ketones or acetonitrile) are unsuitable. The list of possible co-solvents was narrowed down to 1,4-dioxane, methanol, THF and DMSO. 

The first attempts at hydrolysis were conducted with 0.15 mmol (30 mg) of N-benzoylpyrrolidine **3a**, 100 µL of deuterated methanol (as a co-solvent) and 400 µL of a solution of NaOD in D_2_O (40 *w/w* %). The procedure was also done using 100 µL of deuterated 1,4-dioxane(1,4-dioxane-*d*_8_). The samples were heated in a water bath at 37 °C and subjected to TLC. NMR spectra were recorded after 24 and 48 h. However, no hydrolysis product was detected by ^1^H-NMR, and no new product appeared on TLC. Thus, the hydrolysis reaction did not proceed at 37 °C. Furthermore, the NaOD solution and the 1,4-dioxane solution separated into two phases.

Next, the concentration of the base was reduced to 0.4 M and that of the reactant to 0.1 M (i.e., a 4-fold excess of base over starting material). The total reaction volume was 1 mL (100 µL of co-solvent and 900 µL of D_2_O). 

Heating at 50 or 70 °C was applied, and the hydrolysis of **3a** was monitored by TLC analysis. The reaction proceeded at 70 °C. The starting material was no longer detectable after 48 h, and a single spot corresponding to the hydrolysis product (benzoic acid) appeared. However, at 50 °C the starting amide **3a** was still detectable on the TLC plate after 48 h. In order to assess the working range for the other compounds, a similar test was done at 70 °C for *p*-nitro derivative **3c** (no spot of the starting material was detected after 5 h) and *p*-methoxy derivative **3e** (the starting material was still detected after 65 h). Moreover, at the higher temperature, the 1,4-dioxane solution remained monophasic. Therefore, it was decided to work at 70 °C.

**Figure 7:**
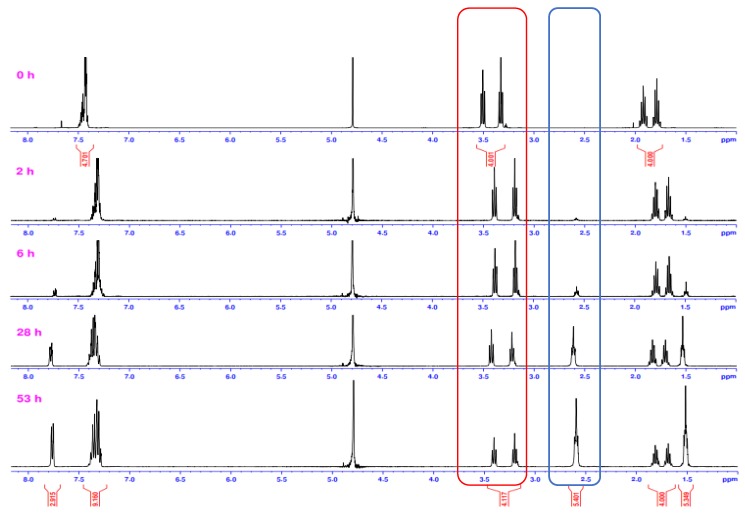
^1^H-NMR monitoring of the hydrolysis of **3a**, with NaOH in D_2_O and 1,4-dioxane-*d*_8_ at 70 °C. The intensity of amide peaks decreased (red box) over time, and product signals appeared (blue box).

In order to follow the progression of the reaction quantitatively, we recorded NMR spectra of hydrolysis reaction mixtures of **3a**–**e** every two min at 70 °C. Rate constants were calculated from the decrease of the integrals of the reactant (Figure 7).

From the ^1^H-NMR integration information we were able to determine the loss of amide over time. Since working with excess deuteroxide anion guarantees pseudo-first-order kinetics, rate constants were calculated using the following first-order equation:

(1) $$\;\frac{d\left[\mathrm{Amide}\right]}{dt}=\;-k\left[Amide\right].\;$$

The integration of this rate equation gives the following equation:

ln[Amide] = −*kt* + ln[Amide]_0_ (2)

where [Amide] represents the molar concentration (M) of amide, *k* represents the reaction constant (s**^−1^**), and ***t*** represents time (**s**). A least-squares plot of the natural logarithm of amide concentration *versus* time gave a straight line whose slope equals −*k*. The initial amide concentration corresponds to the value of the y-intercept.

When either the aromatic protons or the pyrrolidine protons were used as a reference for signal integration, all five pyrrolidine amides **3a**–**e** showed good correlations between concentration and time, and first-order reaction rate constants could be determined (Figure S1). The regression coefficients R^2^ were high for all five compounds **3a**–**e** (Figure S1). The reaction showed Hammett-like behaviour, that is the hydrolysis proceeded faster when an electron-withdrawing group was present at the *para* position of the phenyl ring, and slower when an electron-donating group was present. 

Based on these results, we next examined, the hydrolysis of the bicyclic compounds under the same conditions. In order to hydrolyze compounds **4a** and **4b** it was necessary to increase the proportion of 1,4-dioxane from 10% to 20%. The reaction proceeded smoothly, and the disappearance of the reactants was successfully monitored by NMR. Unfortunately, the bicyclic compounds were not sufficiently soluble under these conditions. Hence, we decided to increase the proportion of co-solvent. The reaction volume was also scaled down from 1 mL to 500 µL. Solvent conditions were modified on the basis of an examination of the hydrolysis of the *p*-NO_2_-substituted pyrrolidine benzamide **3c** (Table 1).

**Figure 8:**
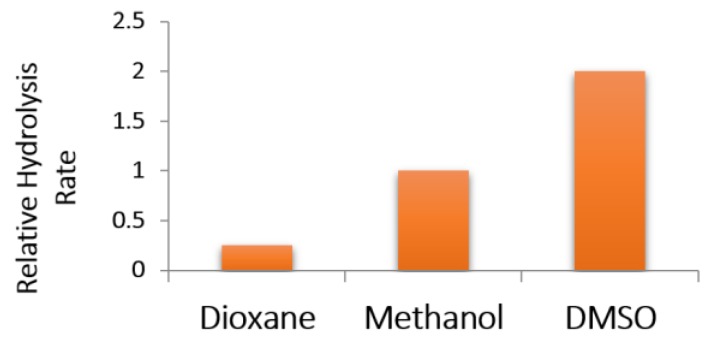
Effect of polarity of the solvent system on the rate of alkaline hydrolysis. Higher polarity of the solvent system accelerates the reaction. The rate of hydrolysis in methanol as a solvent was arbitrarily set at unity (1.0).

The sample containing 75% 1,4-dioxane did not form a homogenous solution even after heating at 70 °C, possibly due to the high NaOD concentration in the water phase. On the other hand, the use of 25% 1,4-dioxane resulted in a low R^2^ value (0.943). Despite these setbacks, it was seen that the reaction proceeds faster in more polar solvent systems. This trend was also seen with other co-solvents (Figure 8). The reaction time was shorter in DMSO-*d*_6_ than in methanol-*d*_4_, which in turn was shorter than in 1,4-dioxane. The reaction was also carried out in THF-*d*_8_, but the compound was not sufficiently soluble even at high temperature. The effect of solvent polarity on the hydrolysis rate can be explained by the fact that the amide bond has a polar nature, and charges develop as the bond is broken. Therefore, more polar solvent systems are better at stabilizing the developing charges in the transition states and the products. Although DMSO is a good solvent, the presence of hydroxide anion can produce the basic dimsyl anion (Na^+ -^CH_2_-SO-CH_3_) from DMSO. Therefore, we focused on 1,4-dioxane and methanol, rather than DMSO.

#### 2.2.2. Alkaline Hydrolysis of Amides in Two Solvents

Finally, the set of conditions, shown in Table 2, was selected for all compounds: 250 µL of co-solvent (1,4-dioxane-*d*_8_ or methanol-*d*_4_), and 250 µL of water (total volume (500 µL)), and 10 equivalents of base with respect to the reactant amide. The reaction was carried out at 70 °C. These conditions provided first-order kinetics with respect to amide concentration.

#### 2.2.3. Alkaline Hydrolysis in Dioxane

**Figure 9:**
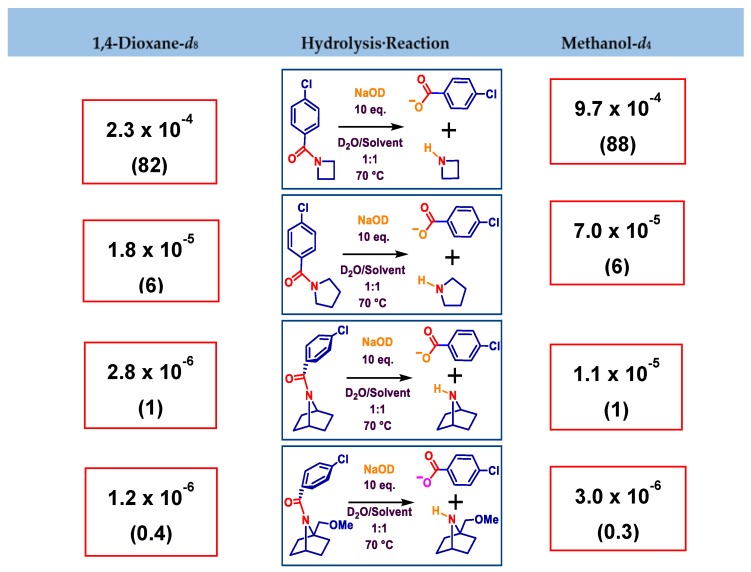
Base-catalyzed hydrolysis rates of **2c, 3c, 4c** and **5c** in two solvent systems (1,4-dioxane and methanol). Values of −*k*_obs_ (s^−1^) are shown. Relative reaction rates are shown in parentheses (referenced to **4c**).

Several compounds were subjected to NaOD-catalyzed hydrolysis in 1,4-dioxane-*d*_8_-D_2_O (1:1) under the conditions, shown in Table 3. The co-solvent was 1,4-dioxane-*d*_8_. The plots in Figure S2 are based on the raw data of selected hydrolysis experiments (Figure 9). Hydrolysis was repeated three times for some of the compounds in order to assess the repeatability of the method. It was found that the error when 1,4-dioxane-*d*_8_ was used as the co-solvent was ±13.8%. Products (carboxylate and amine) were identified by mass spectrometry. NMR monitoring revealed signals corresponding to the reactant and the hydrolysis products in all cases. The ring-opening product of azetidine amide was not detected. As a general trend, base-catalyzed hydrolysis of azetidine amides (**2**) proceeded more rapidly than that of pyrrolidine amides (**3**), which in turn were hydrolyzed faster than unsubstituted bicyclic amides (**4**), while bridgehead-substituted bicycles (**5**) were least reactive (for example, reaction rate: **2a** > **3a** > **4a** > **5a**; **2b** > **3b** > **4b** > **5b**; **2c** > **3c** > **4c** > **5c**). The expected Hammett-like trend was observed: The electron-withdrawing substituent NO_2_ (**c**) on the phenyl moiety accelerated the reaction.

For example, *N*-(*p*-nitrobenzoyl-7-azabicyclo[2.2.1]heptane (**4c**) was hydrolyzed in 1,4-dioxane and D_2_O at a slower rate than the analogous planar monocycle **2c** (6 times faster than **3c**) or the non-planar monocycle **1c** (82–88 times faster than **3c**). Moreover, a bridgehead substituent (**4c**) further slowed the hydrolysis rate (2.3–3.6 times slower than **3c**) (Figure 9).

#### 2.2.4. Alkaline Hydrolysis in Methanol

**Figure 10:**
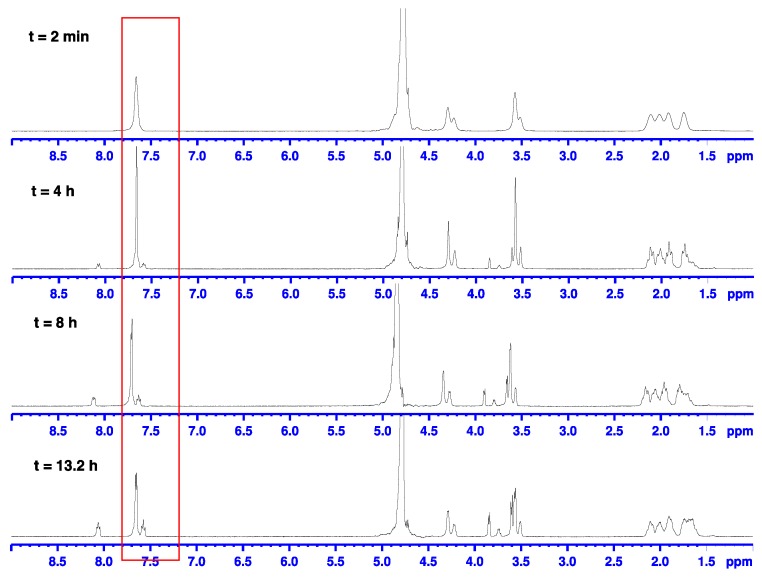
Progress of the hydrolysis of **5b** in methanol-*d*_4_/D_2_O (1:1) at 70 °C, followed by ^1^H-NMR; the intensity of amide peaks decreased, and new product signals appeared as the reaction progressed (red box).

Several compounds were subjected to hydrolysis in methanol under the final conditions, shown in Table 3 (methanol-*d*_4_-D_2_O 1:1). The co-solvent used in this case was methanol-*d*_4_. The following plot shows the ^1^H-NMR spectral change corresponding to the slow consumption of **5b** in methanol (Figure 10). We could not detect intermediate formation of the methyl ester, which may be formed by the attack of methoxide anion on the amide. Hydrolysis was repeated three times for some of the compounds in order to assess the repeatability of the method. It was found that the error of the method when using methanol-*d*_4_ as a co-solvent was ±17.4%. Products were identified by mass spectrometry. Signals corresponding to reactants and hydrolysis products were identified in all cases. The ring-opening product of azetidine amide was not detected. Reactivity followed the same trend as in 1,4-dioxane (reaction rate: **2b** > **3b** > **4b**> **5b**). The reactivity was higher in methanol than in 1,4-dioxane. 

#### 2.2.5. Comparison of Kinetic Data

As shown in Table 3, the same general trend was observed irrespective of the solvent system employed. Azetidine amides (**2**) were the most reactive, followed by pyrrolidine amides (**3**), then unsubstituted bicyclic amides (**4**), and finally bridgehead-substituted bicycles (**5**) (reaction rate: **2b** > **3b** > **4b**> **5b**). Phenyl substitution had the expected effects (according to the inductive effects) in all series of amides.

### 2.3. Computational Studies

#### 2.3.1. Reaction Model

**Scheme 5:**
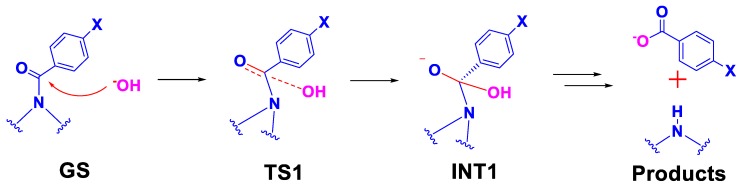
General reaction path for the base-catalyzed hydrolysis of amides.

It was unexpected to find that non-planar amides based on the 7-azabicyclo[2.2.1]heptane scaffold (**4**) showed such poor susceptibility to base-catalyzed hydrolysis, even upon heating, as compared with the corresponding monocyclic amides (**3**). Bridgehead substitution of the 7-azabicyclo[2.2.1]heptane amides (**5**) also decelerated base-catalyzed hydrolysis of the amide as compared with the unsubstituted bicyclic derivative (**4**). It has been established in previous studies on heavy atom isotope effects that formation of the tetrahedral intermediate is rate-determining in the base-catalyzed hydrolysis of formamide (HCONH_2_) (Scheme 5) [27].

While there have been several ab initio and DFT calculation studies of the hydrolysis of planar amides and non-planar amides [28,29,30,31], base-catalyzed hydrolysis of amides has been relatively little studied until recently, Further, most studies have focused on rather simple amides, such as formamide, N-methylacetamide, DMF, and DMA (dimethylacetamide) [32]. Here, we aimed to rationalize the observed reactivity trends by computational studies of our more complex reactants and transition structures. 

**Figure 11:**
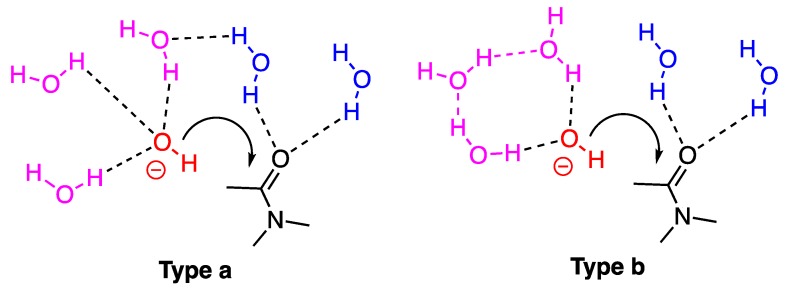
Two hydrogen network patterns involving five H_2_O molecules in the attack of OH anion on the amide carbonyl carbon atom [32].

It is known that that explicit water solvation is crucial for the calculation of amide hydrolysis. Xiong and Zhan [32] showed that incorporation of five implicit water molecules is required, and there are two kinds of hydrogen-bonding networks of water in the vicinity of the hydroxy ion (^−^OH) and amide group in the presence of five explicit water molecules (Figure 11). These two patterns commonly involve activation of the carbonyl group by hydrogen-bonding of two water molecules to the oxygen atom (increasing its electrophilicity) and hydrogen bonding of three water molecules with the oxygen atom of the hydroxy anion (decreasing its nucleophilicity and at the same time decreasing electronic repulsions). There is a difference in the topology of the hydrogen-bonding networks. Type a is more stable than Type b by approximately 1-2 kcal/mol, but finding a TS of Type a in our experimental amide systems **2**–**5** was difficult, probably because the Type a hydrogen network is sensitive to the steric interactions encountered in more complicated amide structures (see the detail in the Experimental Section). Therefore, in the present work, we focused on the hydrogen network Type b, which seems relevant to the present compounds. 

Geometry optimizations for the ground states of hydrated reactants (amide and hydroxide anion) and the transition state for the nucleophilic addition of hydroxide anion to the amide carbonyl group were performed in the presence of explicit water molecules at the B3LYP and M06-2X levels of theory with a combination of two basis sets, 6-31+G(d) and 6-311++G(d, p). Bulk solvation effects (self-consistent reaction field, SCRF) were also incorporated by means of IEFPCM (Polarizable Continuum Model, PCM, using the integral equation formalism variant) and SMD methods in water. Vibrational frequency calculations were performed at the same level of theory. The energies were corrected for the zero-point energies and Gibbs free energy at 25 °C (298.15 K), obtained from frequency calculations. Hereafter, we will focus on the calculation values based on M06-2X level with the SMD solvent model. The calculations at the B3LYP level (see Tables S1–S4) were consistent with the trends obtained in the M06-2X calculations.

#### 2.3.2. Model with Five Explicit Water Molecules

**Scheme 6:**
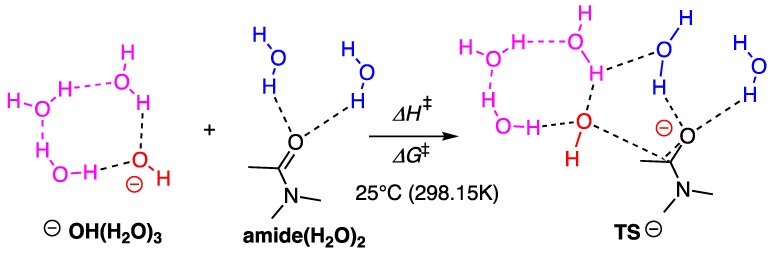
Model of base-catalyzed hydrolysis reaction of amide.

**Figure 12:**
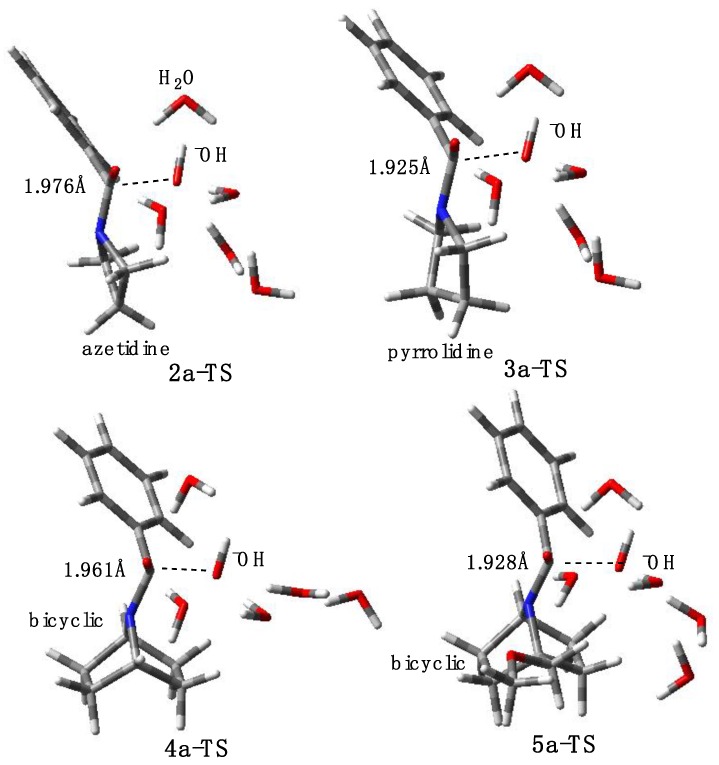
M06-2X/6-31+G(d)-optimized transition structures of hydroxide anion addition to the amide. Distance between the amide carbonyl carbon atom and hydroxide oxygen atom was shown.

Transition structures (TSs) for the hydroxide anion-catalyzed hydrolysis of the amides, including five explicit water molecules were identified with M06-2X//6-31+G(d) (Figure 12). As previously described by Xiong and Zhan [32], we calculated the activation Gibbs energies from the energy difference between the TS structures and the summation of the energies of the two reagents, the amide with two hydrogen-bonded water molecules (amide(H_2_O)_2_) and the hydroxide anion clustered with three water molecules (^−^OH(H_2_O)_3_) (Scheme 6). We also estimated Gibbs free energies by single-point calculation with M06-2X//6-311++G(d,p) on the basis of the M06-2X//6-31+G(d) optimized structures (Table 4). 

The contribution of thermal corrections (entropy term, –*TΔS***^‡^**) was significant. The entropy term (*ΔS***^‡^**) took a large negative value (Table 2), indicating the presence of significant order in the transition structure. Associated water molecules need to rearrange on the surface of the amide, and thus would contribute to this large negative entropy term. A larger free-energy activation barrier was seen for *N*-benzoyl-7-azabicyclo[2.2.1]heptane (**4a**) than for the monocyclic *N*-benzoylpyrrolidine (**3a**). The pyramidal amide, the azetidine derivative **2a** has the smallest activation energy. The order of the magnitude of the Gibbs activation energies (**4a > 3a, 5a > 2a**, Table 4) is essentially consistent with the experimental reactivity (Figure 8 and Table 3), with the exception of the bridgehead-substituted bicyclic amide **5a**, which is expected to have higher activation energy than **4a**. However, we need to make allowance for the simple harmonic oscillator approximation in the thermal energy correction, and also we need to consider that this thermal energy correction is an approximation of the real entropy change in the solvation process.

The enthalpy terms (*ΔH***^‡^**) were underestimated (Table 4), but the order of their magnitudes is also essentially consistent with the experimentally observed hydrolysis rates: **2a** > **3a, 4a, 5a**. This trend is consistent with the trajectory in the TS structures (Figure 12): The shorter the distance between the amide carbonyl carbon atom and hydroxide oxygen atom (i.e., later the TS), the larger the enthalpy term (*ΔH***^‡^**).

Other reaction models, a model with four explicit water molecules and a model with implicit water molecules, were also examined and the order of the energy demand is consistent with that of the present five-water model (the results are described in Supporting Information).

Non-planar amides based on the 7-azabicyclo[2.2.1]heptane scaffold were found to be rather inert to base-catalyzed hydrolysis. The calculated Gibbs free energies are also consistent with the experimental results.

**Figure 13:**
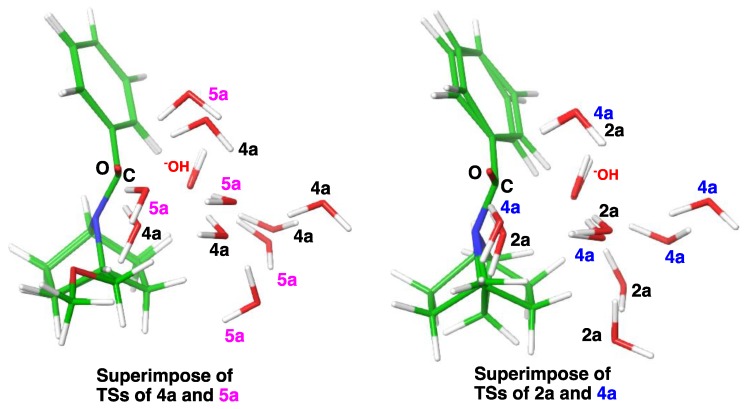
Superimposed TS structures of **4a** and **5a** (left) and **2a and 4a** (right), showing the disrupted water network in bulky **5a** (left, magenta) and **4a** (right, blue).

A close scrutiny of the trajectory in the TS (Figure 12) revealed the optimal trajectory for the hydroxide anion attack on the carbonyl group accompanied with hydrating water molecules. The positions of the hydroxy anion were similar in the respective TS structures. In the bicyclic amides (Figure 13, left), the water molecules in the bridgehead-substituted **5a** were placed a little further from the amide substrate as compared with those in bridgehead-unsubstituted **4a**. A comparison of azetidine **2a** and bridgehead-unsubstituted **4a** also indicated that some of the hydrated waters were placed far from the substrate in the case of **4a** (Figure 13, right). Therefore, these water molecules cannot get close to the substrate, due to greater steric congestion in **4a**, as compared with the azetidine (**2a**). Hydrogen bonding of water molecules stabilized the developing negative charge during the attack of hydroxide anion on the amide carbonyl carbon atom, while the entropy cost compensates for the stabilization. Inefficient hydration is one of the possible reasons that could explain the increase of the activation energy of the bicyclic amides **4** and **5**.

## 3. Materials and Methods 

### 3.1. General Procedures

All analyzed compounds were synthesized from commercially available reagents. All compounds were purified before use by column chromatography on silica gel (spherical, neutral silica gel 60 N (100–210 µm), Kanto). Characterization was done by multiple techniques. ^1^H- (400 MHz) and ^13^C- (100 MHz) NMR spectra were recorded in a 400 MHz Bruker Avance 400 NMR spectrometer at 25 °C. Chemical shifts (δ) are shown in ppm, and coupling constants are given in hertz. Spectral data was obtained using NMR data processing software Brucker TOP-Spin. The NMR probe temperature was calibrated by the temperature-dependent chemical shift difference in ppm between OH proton and CH_2_ proton of ehtyleneglycol [33].

ESI-TOF mass spectra were recorded in a Bruker Daltonics, micrO-TOF-05. Elemental analyses were done by an independent group in this department and were given within a ± 0.4% error range. Melting points were measured with a Yanaco Micro Melting Point Apparatus and are uncorrected.

### 3.2. Synthesis of Amides

All the amide compounds except **5a**–**5c** have been synthesized previously [12] and stock samples were used for the present work. Some compounds among **2a**–**2e**, **3a**–**3e**, and **4a**–**4e** were resynthesized, as described below, including the new compounds **5a**–**5c**.

#### **Synthesis** **of *N*-Benzoylazetidines**

*N-Benzoylazetidine* (**2a**). Azetidine chloride (100 mg, 1.1 mmol) was dissolved in dry CH_2_Cl_2_ (5 mL) and the solution was cooled to 0 °C. DIPEA (0.7 mL, 2.9 mmol) was added, and the mixture was stirred for 10 min. Then, benzoyl chloride (186 µl, 1.6 mmol) was added slowly to the solution, and stirring was continued for 30 min. The ice bath was removed and the reaction mixture was allowed to cool to r.t., then quenched by pouring it into water. The aqueous and organic layers were separated, and the aqueous phase was extracted with dichloromethane (3 × 10 mL). The combined organic phase was washed with 0.5 M HCl, 0.5 M aq. NaHCO_3_ and brine, dried over sodium sulfate, filtered, and evaporated under reduced pressure to afford a yellowish oil. The crude product was purified by open column chromatography (ethyl acetate/DCM, 1:1) to afford **2a** as a transparent oil (130.8 mg, 0.81 mmol, 76%). ^1^H-NMR (400 MHz, CDCl_3_), δ (ppm): 7.616–7.592 (m, 2H), 7.444–7.353 (m, 3H), 4.296–4.191 (m, 4H), 2.352–2.274 (m, 2H). ^13^C-NMR (100 MHz, CDCl_3_), δ (ppm): 170.39, 133.36, 130.93, 128.40, 127.91, 53.46, 49.01. HRMS (ESI-TOF): [M + Na]^+^: Calcd. for C_10_H_11_NNaO^+^: 185.0766. Found: 185.0782.

*N-(p-Chlorobenzoyl)azetidine* (**2b**). Azetidine chloride (100 mg, 1.1 mmol) was dissolved in dry CH_2_Cl_2_ (5 mL) and the solution was cooled to 0 °C. DIPEA (0.7 mL, 2.9 mmol) was added, and the mixture was stirred for 10 min. 4-Chlorobenzoyl chloride (205 µL, 1.6 mmol) was added slowly, and stirring was continued for 30 min. The ice bath was removed and the mixture was allowed to warm to r.t., then quenched by pouring it into water. The aqueous and organic layers were separated, and the aqueous phase was extracted with dichloromethane (3 × 10 mL). The combined organic phase was washed with 0.5 M HCl, 0.5 M aq. NaHCO_3_ and brine, dried over sodium sulfate, filtered, and evaporated under reduced pressure to afford a pale oil. The crude product was purified by open column chromatography (ethyl acetate/DCM, 1:2) to provide **2b** as colorless crystals (171.9 mg, 0.88 mmol, 82%). ^1^H-NMR (400 MHz, CDCl_3_), δ (ppm): 7.587–7.554 (m, 2H), 7.391–7.358 (m, 2H), 4.309–4.199 (m, 4H), 2.386–2.309 (m, 2H). ^13^C-NMR (100 MHz, CDCl_3_), δ (ppm): 169.18, 137.08, 131.76, 129.39, 128.70, 53.51, 49.09. HRMS (ESI-TOF): [M + Na]^+^ Calcd. for C_10_H_10_ClNNaO^+^: 218.0343. Found: 218.0360. Anal. Calcd. for C_10_H_10_ClNO: C, 61.39; H, 5.15; N, 7.16. Found: C, 61.02; H, 5.47; N, 6.96.

*N-(p-Nitrobenzoyl)azetidine* (**2c**). Azetidine chloride (100 mg, 1.1 mmol) was dissolved in dry CH_2_Cl_2_ (5 mL) and the solution was cooled to 0 °C. DIPEA (0.7 mL, 2.9 mmol) was added, and stirring was continued for 10 min. Then, 4-nitrobenzoyl chloride (186 µL, 1.6 mmol) was added slowly, and stirring was continued for 30 min. The ice bath was removed and the mixture was allowed to warm to r.t., then quenched by pouring it into water. The aqueous and organic layers were separated, and the aqueous phase was extracted with dichloromethane (3 × 10 mL). The combined organic phase was washed with 0.5 M HCl, 0.5 M aq. NaHCO_3_ and brine, dried over sodium sulfate, filtered, and evaporated under reduced pressure to give a yellow oil. The crude product was purified by open column chromatography (ethyl acetate/DCM, 1:2) to afford **2c** as a yellow solid (172.6 mg, 0.84 mmol, 78%). ^1^H-NMR (400 MHz, CDCl_3_), δ (ppm): 8.268–8.235 (m, 2H), 7.793–7.760 (m, 2H), 4.311–4.231 (m, 4H), 2.425–2.347 (m, 2H). ^13^C-NMR (100 MHz, CDCl_3_), δ (ppm): 168.00, 139.22, 131.19, 129.01, 123.76, 53.41, 49.32. HRMS (ESI-TOF): [M + Na]^+^ Calcd. for C_10_H_10_N_2_NaO_3_^+^: 229.0585. Found: 229.0585. 

#### **Synthesis** **of *N*-Benzoylpyrrolidines**

*N-Benzoylpyrrolidine* (**3a**). Pyrrolidine (1 mL, 12.2 mmol) was dissolved in dry CH_2_Cl_2_ (5 mL) and the solution was cooled to 0 °C. DIPEA (3.4 mL, 14.5 mmol) was added, and the mixture was stirred for 10 min. Then, benzoyl chloride (1.7 mL, 14.75 mmol) was added slowly, and stirring was continued for 30 min. The ice bath was removed, and the mixture was allowed to warm to r.t., then quenched by pouring it into water. The aqueous and organic layers were separated, and the aqueous phase was extracted with dichloromethane (3 × 10 mL). The combined organic phase was washed with 0.5 M HCl, 0.5 M aqeouse solution of NaHCO_3_ and brine, dried over sodium sulfate, filtered, and evaporated under reduced pressure to give a yellow liquid. The crude product was purified by open column chromatography (ethyl acetate) to afford **3a** as a transparent yellow liquid (1.5530, 8.86 mmol, 73%). ^1^H-NMR (400 MHz, CDCl_3_), δ (ppm): 7.504–7.356 (m, 5H), 3.615 (t, *J* = 6.8 Hz, 2H), 3.381 (t, *J* = 6.6 Hz, 2H), 1.929–1.804 (m, 4H). ^13^C-NMR (100 MHz, CDCl_3_), δ (ppm): 169.35, 137.10, 129.51, 128.00, 126.86, 49.33, 45.93, 26.17, 24.21. HRMS (ESI-TOF) *m*/*z*: [M + Na]^+^ Calcd. for C_11_H_13_NNaO^+^: 198.0890. Found: 198.0872. Anal. Calcd. for C_11_H_13_NO: C, 75.40; H, 7.48; N, 7.99. Found: C, 75.21; H, 7.63; N, 7.97.

*N-(p-Chlorobenzoyl)pyrrolidine* (**3b**). Pyrrolidine (0.5 mL, 6.1 mmol) was dissolved in dry CH_2_Cl_2_ (3 mL) and the solution was cooled to 0 °C. DIPEA (1.7 mL, 7.7 mmol) was added, and the mixture was stirred for 10 min. Then, 4-chlorobenzoyl chloride (1.6164 mL, 7.4 mmol) dissolved in CH_2_Cl_2_ (2 mL) was added slowly, and stirring was continued for 1 h. The ice bath was removed, and the mixture was allowed to warm to r.t., then quenched by pouring it into water. The aqueous and organic layers were separated, and the aqueous phase was extracted with dichloromethane (3 × 10 mL). The combined organic phase was washed with 0.5 M HCl, 0.5 M aq. NaHCO_3_ and brine, dried over sodium sulfate, filtered, and evaporated under reduced pressure to afford a transparent oil. The crude product was purified by open column chromatography (hexane/ethyl acetate) to provide **3b** as a white amorphous solid (444 mg, 2.1 mmol, 35%). m.p.: 62–64 °C. ^1^H-NMR (400 MHz, CDCl_3_), δ (ppm): 7.410 (dd; *J* = 4.3 Hz, 1.8 Hz; 2H), 7. 307 (dd; *J* = 8.8 Hz, 2 Hz; 2H), 3.569 (t, *J* = 6.6 Hz, 2H), 3.349 (t, *J* = 6.6 Hz, 2H), 1.930–1.786 (m, 4H). ^13^C-NMR (100 MHz, CDCl_3_), δ (ppm): 168.48, 135.73, 135.50, 128.63, 128.44, 49.55, 46.25, 26.36, 24.36. HRMS (ESI-TOF): [M + Na]^+^ Calcd. for C_11_H_12_ClNNaO^+^: 232.0500. Found 232.0517. Anal. Calcd. for C_11_H_12_ClNO: C, 63.01; H, 5.77; N, 6.68. Found: C, 62.95; H, 5.78; N, 6.51. 

*N-(p-Nitrobenzoyl)pyrrolidine* (**3c**). Pyrrolidine (0.5 mL, 6.1 mmol) was dissolved in dry CH_2_Cl_2_ (5 mL) at 0 °C. DIPEA (1.7 mL, 7.7 mmol) was added to the solution, and the mixture was stirred for 10 min. Then, 4-nitrobenzoyl chloride (1.1301 g, 7.4 mmol) dissolved in CH_2_Cl_2_ (10 mL) was added slowly, and stirring was continued for 1 h at 0 °C. The ice bath was removed, and the mixture was allowed to warm to r.t., then quenched by pouring it into water. The aqueous and organic layers were separated, and the aqueous phase was extracted with dichloromethane (3 × 30 mL). The combined organic phase was washed with brine, dried over sodium sulfate, filtered, and evaporated under reduced pressure to give a a yellow liquid. The crude product was purified by open column chromatography (ethyl acetate/acetone 1:1) to afford **3c** as a slightly yellow solid (821.6 mg, 3.7 mmol, 61.3%). m.p.: 78–80 °C. ^1^H-NMR (400 MHz, CDCl_3_), δ (ppm): 8.293–8.207 (m, 2H), 7.698–7.665 (m, 2H), 3.676 (t, *J* = 7.0 Hz, 2H), 3.382 (t, *J* = 6.6 Hz, 2H), 2.039–1.887 (m, 4H). ^13^C-NMR (100 MHz, CDCl_3_), δ (ppm): 167.61, 143.19, 128.29, 123.84, 49.61, 46.56, 26.52, 24.50. HRMS (ESI-TOF): [M + Na]^+^ Calcd. for C_11_H_12_N_2_NaO_3_^+^: 244.0774. Found: 244.0742. Anal. Calcd. for C_11_H_12_N_2_O_3_: C, 59.99; H, 5.49; N, 12.72. Found: C, 59.82; H, 5.45; N, 12.65. 

*N-(p-Toluoyl)pyrrolidine* (**3d**). Pyrrolidine (0.5 mL, 6.1 mmol) was dissolved in dry CH_2_Cl_2_ (5 mL) and the solution was cooled to 0 °C. DIPEA (1.7 mL, 7.7 mmol) was added, and the mixture was stirred for 10 min. Then, a solution of *p*-tolyl chloride (975 µL, 7.4 mmol) in CH_2_Cl_2_ (10 mL) was slowly added, and the mixture was stirred for 1 h at 0 °C. The ice bath was removed, and the mixture was allowed to warm to r.t., then quenched by pouring it into water. The aqueous and organic layers were separated, and the aqueous phase was extracted with dichloromethane (3 × 30 mL). The combined organic phase was washed with brine, dried over sodium sulfate, filtered, and evaporated under reduced pressure to provide a yellow liquid. The crude product was purified by open column chromatography (hexane/ethyl acetate 4:1) to afford **3d** as a white solid (654.4 mg, 3.5 mmol, 56.8%). M.p.: 75–76 °C. ^1^H-NMR (400 MHz, CDCl_3_), δ (ppm): 7.438–7.408 (m, 2H), 7.202–7.179 (m, 2H), 3.639 (t, *J* = 7.0 Hz, 2H), 3.439 (t, *J* = 6.6 Hz, 2H), 2.372 (s, 3H), 1.986–1.829 (m, 4H). ^13^C-NMR (100 MHz, CDCl_3_), δ (ppm): 169.93, 139.98, 134.52, 128.93, 127.35, 49.75, 46.30, 26.55, 24.59, 21.50 HRMS (ESI-TOF) *m*/*z*: [M + Na]^+^ Calcd. for C_12_H_15_NNaO^+^: 212.1046. Found 212.1036. Anal. Calcd. for C_12_H_15_NO: C, 76.16; H, 7.99; N, 7.40. Found: C, 75.80; H, 8.09; N, 7.33.

*N-(p-Anisoyl)pyrrolidine* (**3e**). Pyrrolidine (0.5 mL, 6.1 mmol) was dissolved in dry CH_2_Cl_2_ (5 mL) and the solution was cooled to 0 °C. DIPEA (1.7 mL, 7.7 mmol) was added, and the mixture was stirred for 10 min. Then, a solution of 4-nitrobenzoyl chloride (1.2573 g, 7.4 mmol) in CH_2_Cl_2_ (10 mL) was slowly added, and stirring was continued for 1 h at 0 °C. The ice bath was removed, and the mixture was allowed to warm to r.t., then quenched by pouring it into water. The aqueous and organic layers were separated, and the aqueous phase was extracted with dichloromethane (3 × 30 mL). The combined organic phase was washed with brine, dried over sodium sulfate, filtered, and evaporated under reduced pressure to provide a yellow liquid. The crude product was purified by open column chromatography (*n*-hexane/ethyl acetate 1:1) to afford **3e** as a pale-brown solid (808.3 mg, 3.9 mmol, 65%). m.p.: 73.5–75.5 °C. ^1^H-NMR (400 MHz, CDCl_3_), δ (ppm): 7.515 (ddd, *J* = 9.2, 2.4, 2 Hz, 4H), 3.832 (s, 3H), 3.634 (t, *J* = 6.4 Hz, 2H), 3.476 (t, *J* = 6.2 Hz, 2H), 1.966–1.851 (m, 4H). ^13^C-NMR (100 MHz, CDCl_3_), δ (ppm): 169.56, 160.93, 129.63, 129.29, 113.55, 55.45, 49.90, 46.44, 26.64, 24.59. HRMS (ESI-TOF) *m*/*z*: [M + Na]^+^ Calcd. for C_12_H_15_NNaO_2_^+^: 228.0995. Found 228.0993. Anal. Calcd. for C_12_H_15_NO_2_: C, 70.22; H, 7.39; N, 6.82. Found: C, 70.01; H, 7.48; N, 6.76.

#### **Synthesis** **of *N*-Benzoylazetidines**

*N-Benzoyl-7-azabicyclo[2.2.1]heptane* (**4a**). *Trans*-4-aminocyclohexyl *p*-toluensulfonate hydrobromide (1 g, 7.5 mmol) was dissolved in ethanol (110 mL) and water (30 mL), and then NaOH 1 M (25 mL) was added. The mixture was stirred at room temperature for 20 h, and then quenched by adding HCl 4 M in 1,4-dioxane (5 mL). Stirring was continued for 20 min at r.t., and the mixture was evaporated under reduced pressure. NaOH 10% (10 mL) was added to the residue, and free amide was extracted with ether (3 × 30 mL). This solution was evaporated, and the residue was redissolved in dry CH_2_Cl_2_ (30 mL). DIPEA (2 mL, 9.3 mmol) was added to the resulting solution, and the mixture was stirred for 10 min at 0 °C. Then, benzoyl chloride (1.1 mL, 9.1 mmol) was slowly added. Stirring was continued for 30 min. The ice bath was removed, and the mixture was allowed to warm to r.t. Stirring was continued for 4 h, and then the mixture was quenched by pouring it into water. The aqueous and organic layers were separated, and the aqueous phase was extracted with dichloromethane (3 × 50 mL). The combined organic phase was washed with brine, dried over sodium sulfate, filtered, and evaporated under reduced pressure to give a yellow oil. The crude product was purified by open column chromatography (CH_2_Cl_2_/ethyl acetate 9:1) to afford **4a** as a white solid (52.0 mg, 35%). ^1^H-NMR (400 MHz, CDCl_3_), δ (ppm): 7.813–7.696 (m, 2H), 7.506–7.288 (m, 3H) 4.613–4.460 (m, 1H), 4.121–3.900 (m, 1H), 2.239–1.240 (m, 8H). ESI-HRMS (*m*/*z*): Calculated for C_13_H_15_NNaO^+^ [M + Na]^+^: 225.1079. Found: 225.1081.

*N-(p-Toluoyl)-7-azabicyclo[2.2.1]heptane* (**4d**). *Trans*-4-aminocyclohexyl *p*-toluensulfonate hydrobromide (1 g, 7.5 mmol) was dissolved in ethanol (110 mL) and water (30 mL), and then NaOH 1 M (25 mL) was added. The solution was stirred at room temperature for 20 h, and then quenched by adding concentrated HCl 4 M (5 mL). Stirring was continued for 20 min at r.t., and then the solution was evaporated under reduced pressure. NaOH 10% (10 mL) was added to the residue. Free amide was extracted with ether (3 × 30 mL), then HCl in 1,4-dioxane (1 mL) was added, and the mixture was evaporated. The residue was redissolved in dry CH_2_Cl_2_ (30 mL). DIPEA (2 mL, 9.3 mmol) was added to the resulting solution, and the mixture was stirred for 10 min at 0 °C. Then, *p*-tolyl benzoyl chloride (1.6 mL, 9.1 mmol) was slowly added. Stirring was continued for 30 min. The ice bath was removed, and the mixture was allowed to warm to r.t., and further stirred for 4 h, then quenched by pouring it into water. The aqueous and organic layers were separated, and the aqueous phase was extracted with dichloromethane (3 × 30 mL). The combined organic phase was washed with brine, dried over sodium sulfate, filtered, and evaporated under reduced pressure to give a yellow oil. The crude product was purified twice by open column chromatography (*n*-hexane/ethyl acetate (1:1) and dichloromethane/ethyl acetate (9:1)) to afford **4d** as a yellow solid. Recrystallization afforded transparent crystals (238.6 mg, 11%). M.p.: 105–107 °C. ^1^H-NMR (400 MHz, CDCl_3_), δ (ppm): 7.449 (d, *J* = 8 Hz, 2H), 7.188 (d, *J* = 8Hz, 2H), 4.720 (br. s, 1H), 4.148 (br. s, 1H), 2.377 (s, 3H), 1.899–1.810 (s, 4H), 1.508–1.466 (m, 4H). ^13^C-NMR (100 MHz, CDCl_3_), δ (ppm): 169.03, 140.72, 133.50, 128.96, 127.97, 77.36, 30.60, 28.87, 21.56. ESI-HRMS: Calculated for C_14_H_17_NNaO^+^ [M + Na]^+^: 238.1202. Found: 238.1217. Anal. Calcd. for C_14_H_17_NO_2_: C, 78.10; H, 7.96; N, 6.51. Found: C, 77.76; H, 7.90; N, 6.58. 

#### **Synthesis** **of *N*-Benzoyl-1-(Methoxymethyl)-7-azabicyclo[2.2.1]heptanes**

*N-Benzoyl-1-(Methoxymethyl)-7-azabicyclo[2.2.1]heptane* (**5a**). Boc-protected 1-(methoxymethyl)-7-azabicyclo[2.2.1]heptane (114.6 mg, 0.47 mmol) was dissolved in a 1:1 mixture of dry CH_2_Cl_2_ (1 mL) and TFA (1 mL), and the solution was stirred at room temperature for one h. The solvent was removed in vacuo, and the residue was dissolved in CH_2_Cl_2_. The resulting solution was evaporated again (a total of three times). Finally, the residue was dissolved in dry CH_2_Cl_2_ (5 mL) and DIPEA (130 µl, 0.61 mmol) was added to the solution. The mixture was stirred for 10 min at 0 °C, then benzoyl anhydride (170 µL, 0.9 mmol) was slowly added. Stirring was continued for 30 min. The ice bath was removed, and the mixture was allowed to warm to r.t., then quenched by pouring it into water. The aqueous and organic layers were separated, and the aqueous phase was extracted with dichloromethane (3 × 10 mL). The combined organic phases were washed with brine, dried over sodium sulfate, filtered, and evaporated under reduced pressure to give a yellow liquid. The crude product was purified by open column chromatography (CH_2_Cl_2_/ethyl acetate 9:1) to afford **5a** as a white solid (520.0 mg, 35%). M.p.: 53–55°C. ^1^H-NMR (400 MHz, CDCl_3_), δ (ppm): 7.524–7.512 (m, 2H), 7.437–7.342 (m, 3H), 4.225 (s, 2H), 4.106 (t, *J* = 5 Hz, 1H), 3.425 (s, 3H), 2.013–1.932 (m, 2H), 1.867–1.771 (m, 2H), 1.728–1.667 (m, 2H), 1.487–1.425 (m, 2H). HRMS (ESI-TOF): [M + Na]^+^ Calcd. for C_15_H_19_NNaO_2_^+^ [M + Na]^+^: 268.1308. Found: 268.1312. 

*N-(p-Chlorobenzoyl)-(1-(methoxymethyl)-7-azabicyclo[2.2.1]heptanes* (**5b**). To a solution of the corresponding Boc-protected 1-(methoxymethyl)-7- azabicyclo[2.2.1]heptane (41.2 mg) in dry CH_2_Cl_2_ (2 mL) was added 3 mL of TFA. The solution was stirred at room temperature for 2 h The organic solvent was evaporated, and the residue was dissolved in 10 mL of CH_2_Cl_2_. Triethylamine (93.6 µL) was added at 0 °C, followed by *p*-chlorobenzoyl chloride (58.8 mg). The reaction mixture was stirred for 2 h at 0 °C, and then poured into saturated aqueous NaHCO_3_. The whole was extracted with CH_2_Cl_2_ (30 mL × 3)_._ The combined organic layer was dried over Na_2_SO_4_, and the solvent was evaporated. Column chromatography (*n*-hexane:ethyl acetate = 4:1) of the residue gave **5b** (47.8 mg, 77%). ^1^H-NMR (400 MHz, CDCl_3_), δ (ppm): 7.489 (2H, d, *J* = 8.4 Hz), 7.356 (2H, d *J* = 8.0 Hz), 4.198 (2H, s), 4.087 (1H, m), 3.423 (3H, s), 2.076–1.571 (6H, m), 1.568–1.369 (2H, m). ^13^C-NMR (100 MHz, CDCl_3_), δ (ppm): 169.11, 136.37, 135.45, 129.20, 128.43, 73.91, 68.03, 61.37, 59.34, 32.65, 29.59. HRMS (ESI-TOF): [M + Na]^+^ Calcd. for C_15_H_18_ClNNaO_2_^+^ [M + Na]^+^: 302.0918. Found: 302.0924. Anal. Calcd. for C_15_H_18_ClNO_2_: C, 64.40; H, 6.49; N, 5.01. Found: C, 64.27; H, 6.52; N, 4.92. 

*N-(p-Nitrobenzoyl)-(1-(Methoxymethyl)-7-azabicyclo[2.2.1]heptanes* (**5c**). Boc-protected 1-(methoxymethyl)-7-azabicyclo[2.2.1]heptane (119.3 mg, 0.49 mmol) was dissolved in a 1:1 mixture of dry CH_2_Cl_2_ (1 mL) and TFA (1 mL), and the solution was stirred at room temperature for one h. The solvent was removed in vacuo, then the residue was dissolved in CH_2_Cl_2_ and this solution was evaporated again (a total of three times). Finally, the residue was dissolved in dry CH_2_Cl_2_ (5 mL), DIPEA (132 µL, 0.63 mmol) was added to it, and the mixture was stirred for 10 min at 0 °C. Then, *p*-nitrobenzoyl chloride (91.70 mg, 0.6 mmol) was slowly added, and stirring was continued for 30 min. The ice bath was removed, and the mixture was allowed to warm to r.t. Stirring was continued for 5.5 h, and then the mixture was quenched by pouring it into water. The aqueous and organic layers were separated, and the aqueous phase was extracted with dichloromethane (3 × 10 mL). The combined organic phase was washed with brine, dried over sodium sulfate, filtered, and evaporated under reduced pressure to give a a yellow liquid. The crude residue was purified by open column chromatography (*n*-hexane/ethyl acetate 1:1) to afford **5c** as a white solid (63.9 mg, 45%). m.p.: 140–142 °C. ^1^H-NMR (400 MHz, CDCl_3_), δ (ppm): 8.256 (dd, *J* = 2 Hz, 6.8 Hz, 2H), 7.685 (dd, *J* = 2 Hz, 6.8 Hz, 2H), 4.178 (s, 2H), 4.010 (t, *J* = 4.4 Hz, 1H), 3.419 (s, 3H), 2.003–1.961 (m, 2H), 1.873–1.783 (m, 2H), 1.750–1.689 (m, 2H), 1.549–1.487 (m, 2H). ^13^C-NMR (100 MHz, CDCl_3_), δ (ppm): 167.52, 148.90, 143.12, 128.68, 123.74, 73.64, 68.66, 61.24, 59.46, 32.79, 29.71. HRMS (ESI-TOF): [M + Na]^+^ Calcd. for C_15_H_18_N_2_NaO_4_^+^ [M + Na]^+^: 313.1159. Found: 313.1169. Anal. Calcd. for C_15_H_18_N_2_O_4_: C, 62.06; H, 6.25; N, 9.65. Found: C, 61.92; H, 6.28; N, 9.63. 

### 3.3. Kinetic Studies

Kinetic data were collected by recording the ^1^H-NMR (400 MHz) spectra at 2- min intervals. Data were obtained at 70.0 °C under a N_2_ flow of 400 L/h. Deuterated solvents were used for all measurements: Deuterium oxide, 99.9% D from Wako; methanol-d_4_, 99.8% D from Kanto Chemical Co. Inc.; 1,4-dioxane-d_8_, 99% D from Cambridge Isotope Laboratories, Inc.; and sodium deuteroxide, 40% wt. % in D_2_O, 99.5% D from Sigma-Aldrich, Co. Parameters were set using the reaction solution at room temperature. Reactions were initiated by raising the temperature of the NMR machine. Spectral data were then recorded for at least one half-life. Hydrolysis experiments were repeated three times. Hydrolysis products were identified by comparison of the ^1^H-NMR data with those of authentic samples, or by MS-ESI. Pseudo-first-order rate constants (*k_obs_*) were evaluated by a linear least-squares fitting of a plot of the logarithm of reactant concentration *versus* time.

### 3.4. Computational Studies

Computational studies were carried out using the Gaussian 09 software package [34]. Geometry optimizations for the model molecules were performed at the M06-2X/6-31+G(d) level with the bulk solvent model SMD (solvent = water) together with explicit water molecules. Vibrational frequency calculations were performed at the same level of theory. Optimized geometries were verified by frequency calculations as minima (zero imaginary frequencies) or transition structures (TS, a single imaginary frequency) (the coordinates, frequency and thermodynamic values are shown in Supporting Information). Intrinsic reaction coordinate (IRC) computations of the transition structures verified the reactants and products in the case of simple amide (formamide and acetamide (data not shown)). In the cases of more realistic substrates (**2a**–**5a**), particularly in combination of the solvent model, the IRC calculations were unsuccessful. In all the transition states the validity of transition state structures was confirmed by inspecting the direction of vibration of the negative frequency, which matched the trajectory of the nucleophilic attack of the hydroxy anion onto the carbonyl carbon atom. Geometry minimization of the transition structures lead to the hydroxide-addition adducts, which also supported the validity of the transition state structures, which represented the trajectory of the nucleophilic attack of the hydroxy anion. We also estimated Gibbs free energies by single-point calculations (frequency calculations) with M06-2X//6-311++G(d,p) for the M06-2X//6-31+G(d)-optimized structures. In these single-point frequency calculations, a single negative frequency was found respectively, which also corresponded to the trajectory of the nucleophilic attack of the hydroxy anion onto the carbonyl carbon atom. The energies were corrected for zero-point energies and Gibbs free energy at 25 °C (298.15 K), obtained from frequency calculations. Calculations at the B3LYP level showed similar trends to the M06-2X calculations.

Identification of the transition states structures of the attack of the hydroxide anion to the carbonyl carbon atom of the amide group was carried out in the following multiple procedures: The transition state structures of the attack of the hydroxide anion to the carbonyl carbon atom of the amide were first identified in the absence of the explicit water molecules (data not shown). Addition of explicit water molecules in the arrangement similar to Types a and b (Figure 11) [32], followed by geometry optimization (OPT = TS). Or we reproduced the TSs of the hydrolysis of formamide and acetamide in the presence of four and five water molecules, respectively, which were consistent with the two hydrogen network models (Types a and b, Figure 11), reported in the previous literature [32]. Then the amide functionality was morphed into the realistic amide substrates (**2a**–**5a**). The initial minimum conformation of the neutral amide substrates (**2a**–**5a**) were obtained by conformational search in Marcomodel software (Schrödinger, LLC, New York, NY, USA), followed by optimization with the DFT methods. The Type a arrangement of waters (Figure 11) did not converge to the optimization or transformed to the Type b arrangement of water molecules. Therefore, we discussed the activation energies on the basis of the Type b hydrogen network model, while the Type **a** hydrogen network model might be the energy minimum (see the main text). 

The change of Gibbs free energy for the reaction was calculated on the basis of the reaction model, shown in Scheme 6. The results of the present calculations (Table 4) were reasonable and reliable, because the present calculations gave the activation energies of similar magnitude to the cases of simple amide: The calculated activation energies of the present amides **2a**–**5a** were in similar magnitude to the calculated and experimental Gibbs free energy change of hydroxide anion-catalyzed hydrolysis of formamide, 21–22 kcal/mol [32], (see also Table 4, footnote a). 

## 4. Conclusions

Herein we measured and compared the kinetics of base-catalyzed hydrolysis of non-planar *N*-benzoyl-7-azabicyclo[2.2.1] heptane amides (**4**) and related compounds (**2**, **3** and **5**) under pseudo-first-order conditions at 70 °C. Excess sodium deuteroxide was used as the base in two different solvent systems (methanol or 1,4-dioxane in a 1:1 D_2_O solution). Reaction progress was monitored by ^1^H NMR spectroscopy. Unexpectedly we found that 7-azabicyclo[2.2.1]heptane amides (**4**) were resistant to base-catalyzed hydrolysis. As a general trend, independently of whether methanol or 1,4-dioxane was used as a co-solvent, it was found that the reactivity of nitrogen-pyramidal azetidine amides (**2**) was greater than that of the planar pyrrolidine amides (**3**), followed by unsubstituted bicyclic amides (**4**), while bridgehead-substituted bicycles (**5**) were the least reactive (reaction rate: **2a** > **3a** > **4a** > **5a**, etc.). Phenyl substituents showed the expected electronic trends. We also executed DFT calculations of the rate-determining process, addition of the hydroxide anion to the amide carbonyl group, and found that the experimental kinetic data is consistent with the magnitude of the calculated Gibbs free energies of activation. Our results confirm the stability of the 7-azabicyclo[2.2.1]heptane amides at least at 37 °C. This is important, because it implies that the 7-azabicyclo[2.2.1]heptane amide scaffold is available for practical molecular design, e.g., as an amino acid surrogate.

## Figures and Tables

**Table 1:** Effect of variations of co-solvent proportions on the hydrolysis rate of **3c**.

1,4-Dioxane-*d*_8_/D_2_O (*v/v*%)	3c	NaOD	Temperature	*−k_obs_* (s^−1^)
25/75	0.05 mmol	0.5 mmol	70 °C	3.0 × 10^−4^
50/50	0.05 mmol	0.5 mmol	70 °C	9.6 × 10^−5^
75/25	0.05 mmol	0.5 mmol	70 °C	2.3 × 10^−6^

**Table 2:** Final conditions for alkaline hydrolysis of amide compounds.

Starting Amide	NaOD 40% wt.	D_2_O	Co-solvent	Temperature
0.05 mmol	0.5 mmol	250 µL	250 µL	70 °C

**Table 3:** Base-catalyzed hydrolysis rates (−*k*_obs_ in M^−1^s^−1^) of amides **2x**–**5x** (**x = a**–**e**) in two solvent systems.^a^ Average values are shown where possible (see footnotes). Relative reaction rates (referenced to **4**) are shown in parentheses.

	1,4-Dioxane-D_2_O (1:1), NaOD, 70 °C ^a^				Methanol-D_2_O (1:1), NaOD, 70 °C ^b^			
x=	2x	3x	4x	5x	2x	3x	4x	5x
**a (H)**	2.1 × 10^−5^ (5.8)	7.8 × 10^−6^ (2.2)	3.6 × 10^−6^ (1)	6.5 × 10^−7^ (0.2)	1.1 × 10^−4^	2.9 × 10^−5^	ND	ND
**b (Cl)**	2.3 × 10^−4^ (82.1)	1.8 × 10^−5^ (6.4)	2.8 × 10^−6^ (1)	1.2 × 10^−6^ (0.4)	9.7 × 10^−4^ (88.2)	7.0 × 10^−5^ (6.4)	1.1 × 10^−5^(1)	3.0 × 10^−6^ (0.3)
**c (NO_2_)**	9.9 × 10^−4^ (33.0)	1.0 × 10^−4^ (3.3)	3.0 × 10^−5^ (1)	3.7 × 10^−5^ (1.2)	6.5 × 10^−3^ (92.9)	4.2 × 10^−4^ (6.0)	7.0 × 10^−5^(1)	4.3 × 10^−5^ (0.6)
**d (Me)**	ND	4.1 × 10^−6^	NA	NE	ND	1.7 × 10^−5^ (4.2)	4.1 × 10^−6^(1)	NE
**e (MeO)**	ND	4.8 × 10^−6^	NE	NE	ND	1.9 × 10^−5^	NE	NE

^a^ Error estimation: ±13.8% (in the 1,4-dioxane system). (**b**) Error estimation: ±17.4% (in the methanol system). ND = not determined; NA = not available (due to the solubility problem); NE = not executable (due to very slow reaction).

**Table 4:** Calculated free energy barrier for the formation of the transition state at 25 °C (298.15 K) at the M06-2X/6-31+G(d) and M06-2X/6-311++G(d, p) levels, considering five water molecules. Solvent effect is SMD (solvent = water) ^a^.

Compound	*ΔH*^‡^ kcal/mol	−*TΔS*^‡^ kcal/mol	*ΔS*^‡^ cal/(mol·K)	*ΔG*^‡^_25 °C_ kcal/mol
M06-2X/6-31+G(d) SMD=water ^b^				
**2a**	2.51	+17.38	−58.29	19.89
**3a**	5.03	+17.03	−57.12	22.06
**4a**	6.80	+16.57	−55.58	23.37
**5a**	4.66	+17.95	−60.20	22.61
M06-2X/6-311++G(d,p) SMD=water ^c^				
**2a**	4.26	+16.16	−54.20	20.42
**3a**	7.13	+16.03	−53.76	23.16
**4a**	8.80	+15.76	−52.86	24.56
**5a**	7.50	+15.44	−51.79	22.94

^a^ Calculation and experimental Gibbs free energy of hydroxide anion-catalyzed hydrolysis of formamide was 21–22 kcal/mol [32]. ^b^ Full optimizations. ^c^ Single-point calculations.

## Supplementary Materials

The following are available online, Supporting Information: Raw kinetic data (Figures S1 and S2), additional calculation data (Tables S1–S5), calculation coordinates, NMR spectra (Figures S3–S5).
