# Supplementary material for: Unexpected Resistance to Base-Catalyzed Hydrolysis of Nitrogen Pyramidal Amides Based on the 7-Azabicyclic[2.2.1]heptane Scaffold
Source: Molecules. 2018 Sep 15;23(9):2363. doi: 10.3390/molecules23092363 (PMC6225387; doi:10.3390/molecules23092363)

Supporting Information

Unexpected Resistance to Base-catalyzed Hydrolysis of Nitrogen Pyramidal Amides Based on the 7-Azabicyclic[2.2.1]heptane Scaffold

Diego Antonio Ocampo Gutiérrez de Velasco,[1] Aoze Su,[1] Luhan Zhai,[1]

Satowa Kinoshita,[1,2] Yuko Otani,[1] Tomohiko Ohwada[1,*]

[1] Laboratory of Organic and Medicinal Chemistry, Graduate School of Pharmaceutical Sciences, University of Tokyo,

7-3-1 Hongo, Bunkyo-ku, Tokyo 113-0033, Japan

2. Deparment of Chemistry, St John's College, University of Cambridge,

St John's Street, Cambridge CB2 1TP, United Kingdom

E-mail: ohwada@mol.f.u-tokyo.ac.jp

Table of contents

1. Measurement of Kinetics of the base-catalyzed hydrolysis

2. Results of Calculations of Different Model

3. Caluculation Coordinates, Frequecy, thermal values of Transition States

4. Spectra data of new compounds

1. **Measurement of Kinetics of the base-catalyzed hydrolysis**

Kinetic data of 2a-e, 3a-e, 4a-4c, 5a-c

Figure S1. Hydrolysis in 1,4-Dioxane-d_8-D_2O (50:50)-NaOD 70°C

2a (X=H)

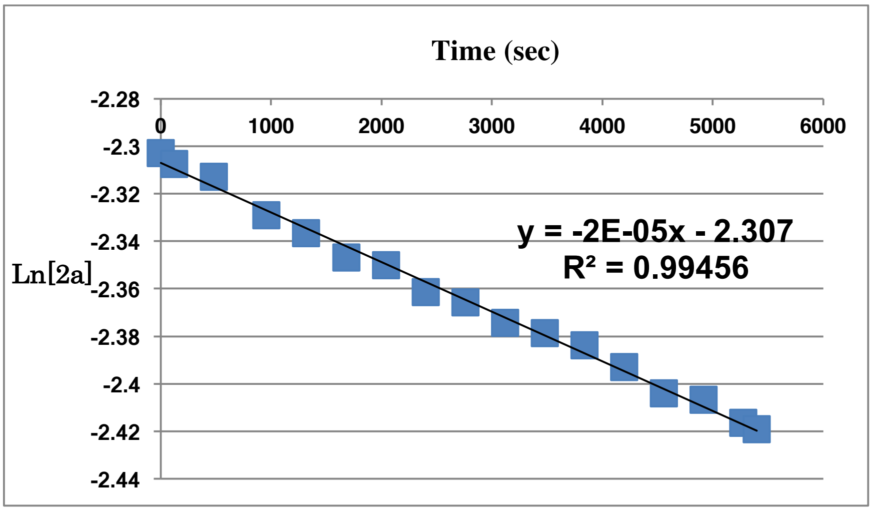

2b (X=Cl)

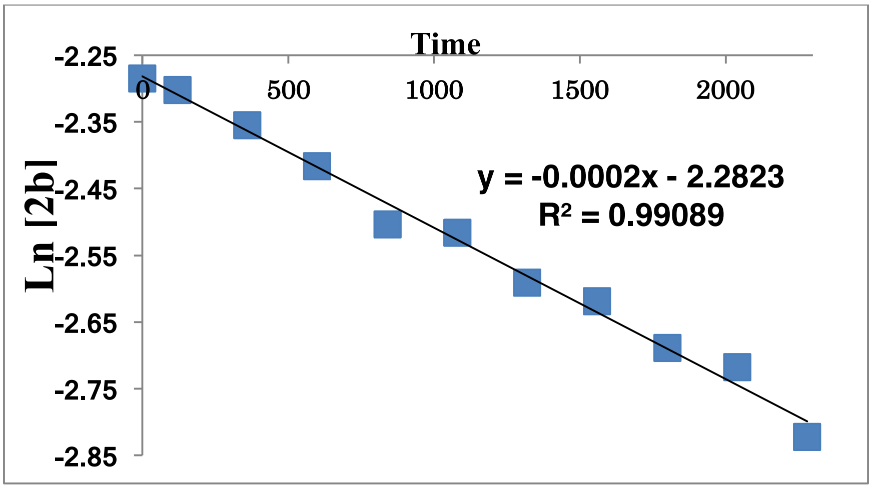

2c (X=NO2)

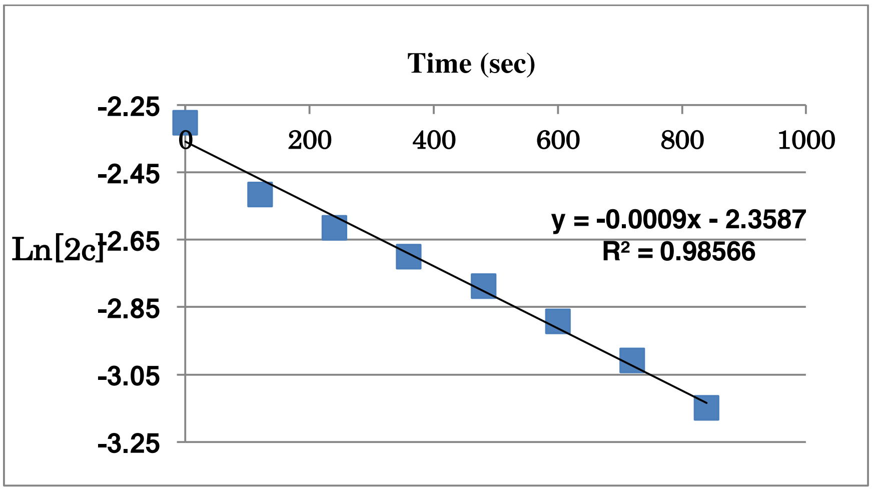

3a (X=Me)

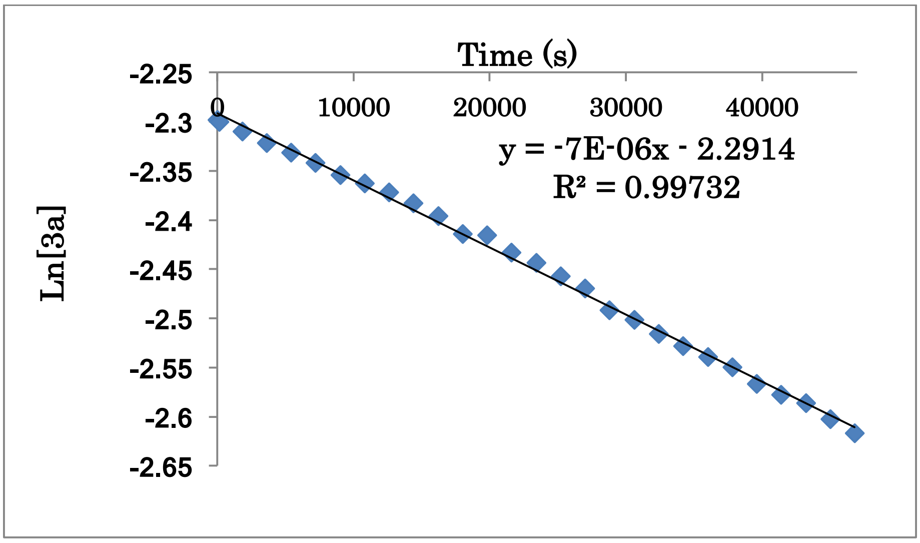

3b (X=Cl)

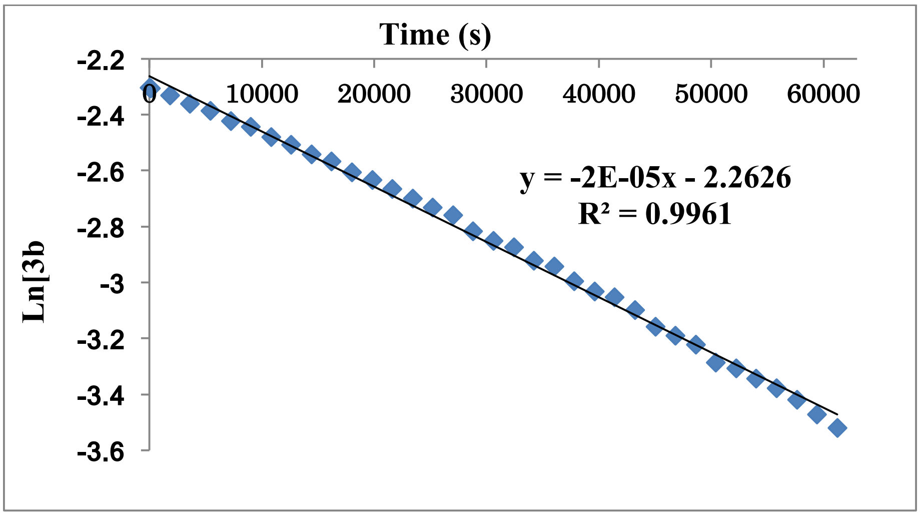

3c (X=NO2)

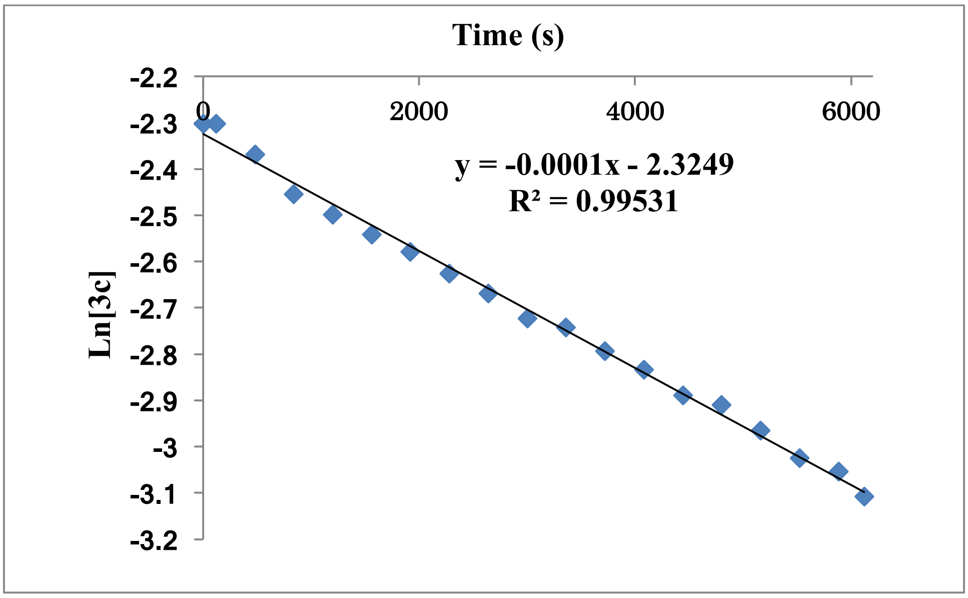

3d (X=Me)

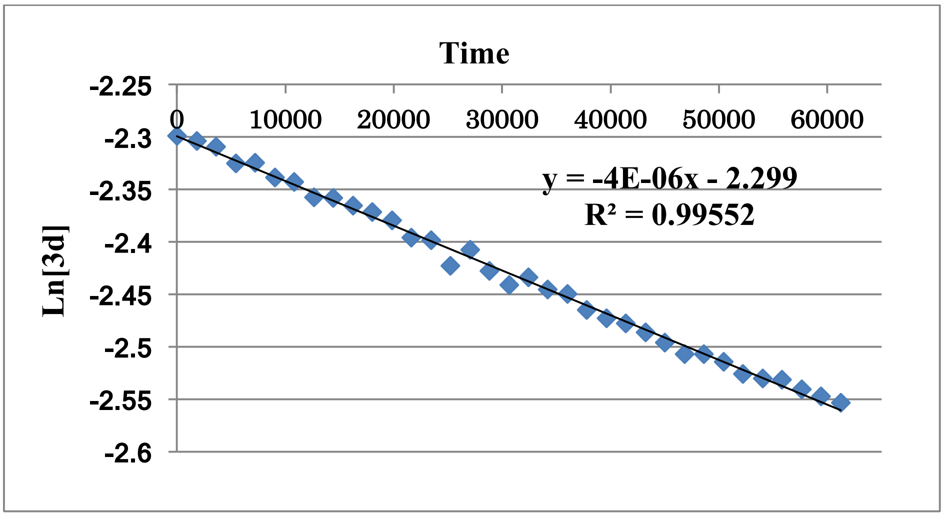

3e (X=OMe)

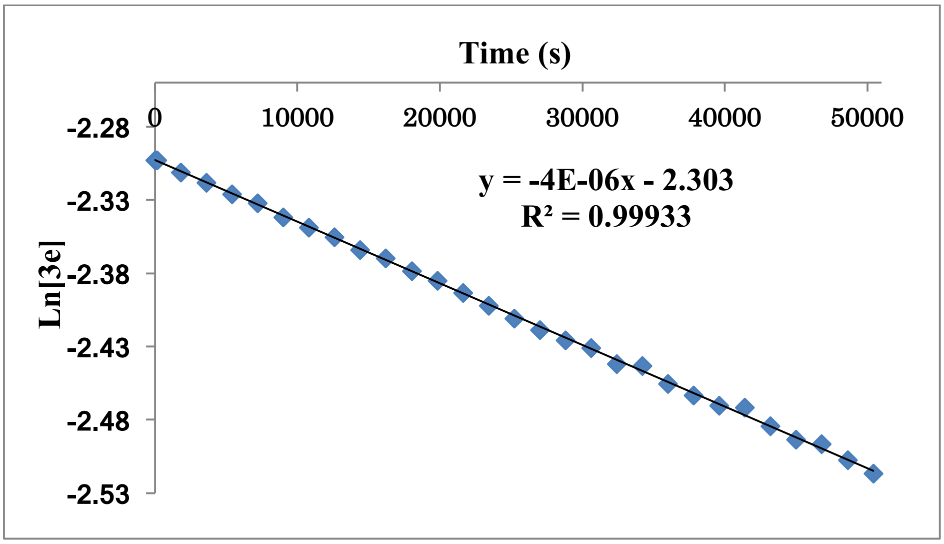

4a (X=H)

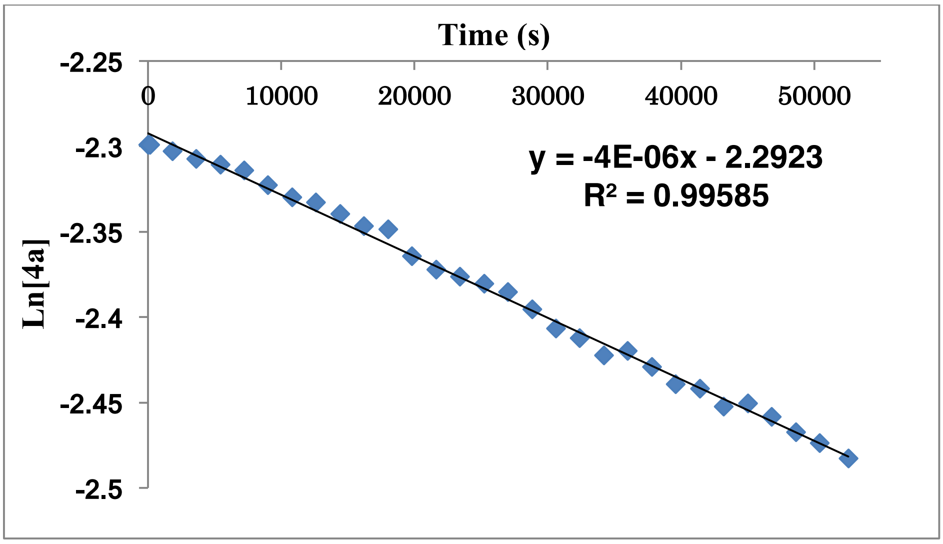

4b (X=Cl)

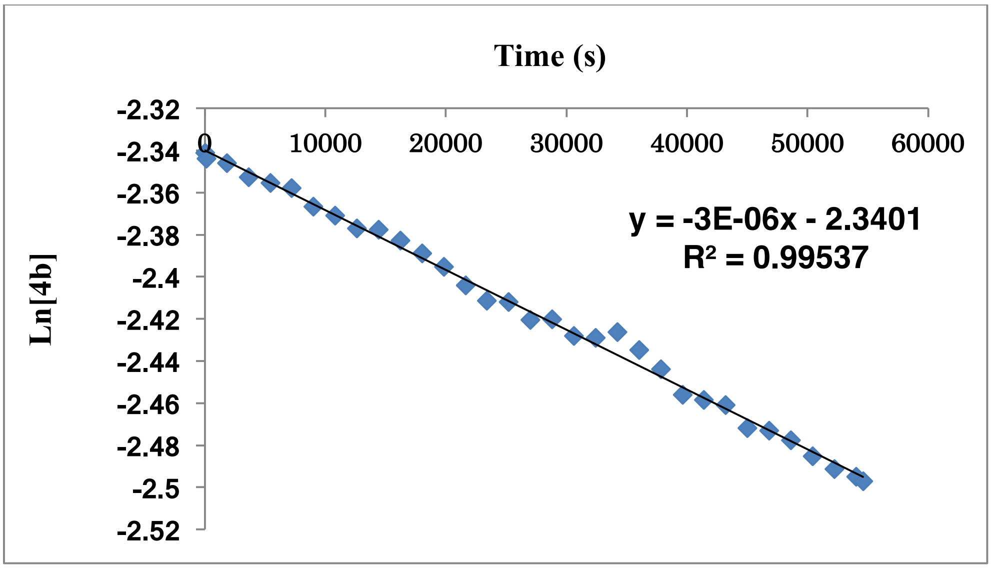

4c (X=NO_2)

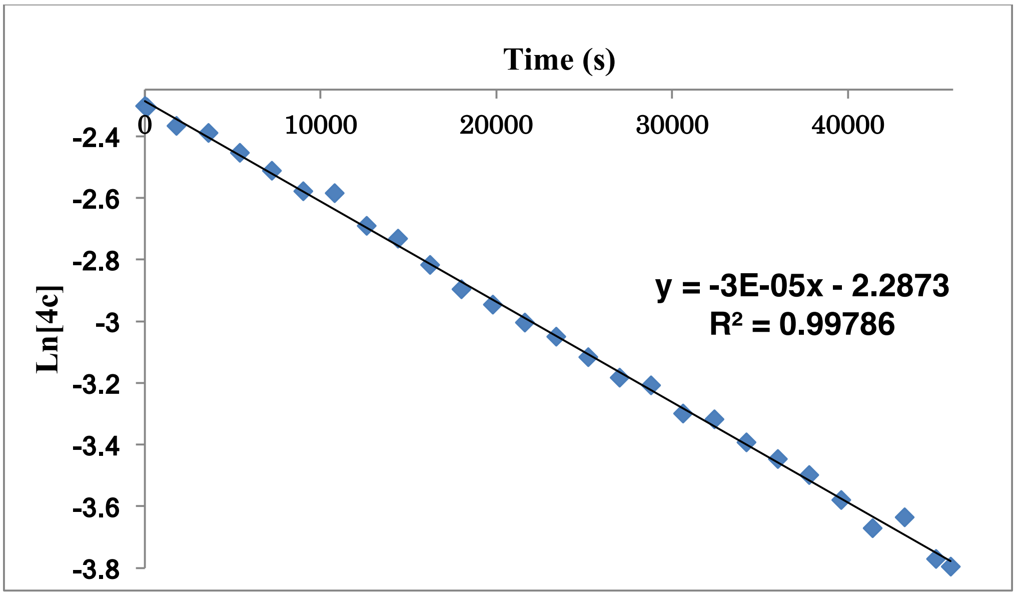

5a (X=H) (20% 1,4-dioxane-d_8-80% D_2O)

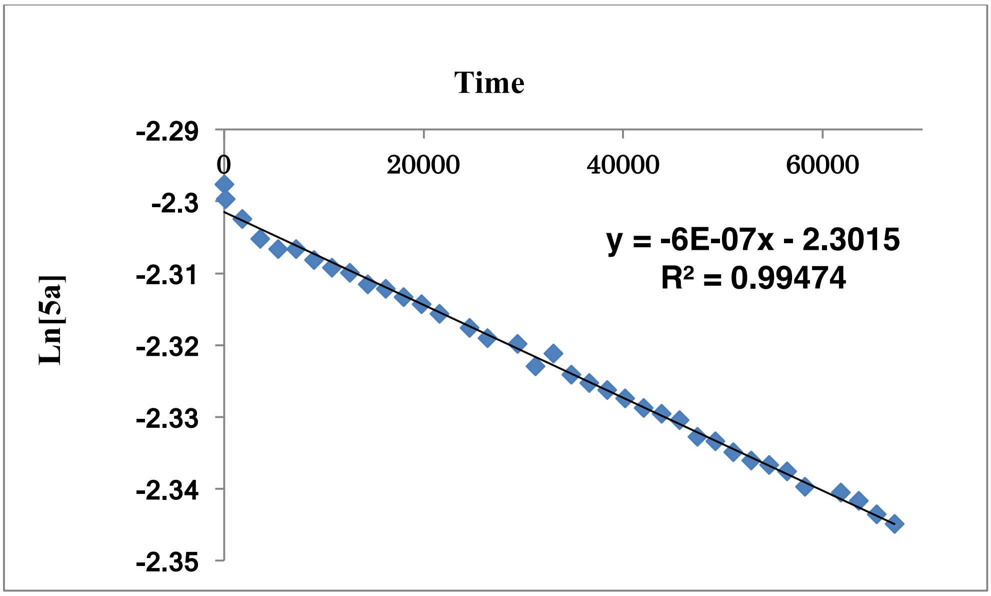

5b (X=Cl)

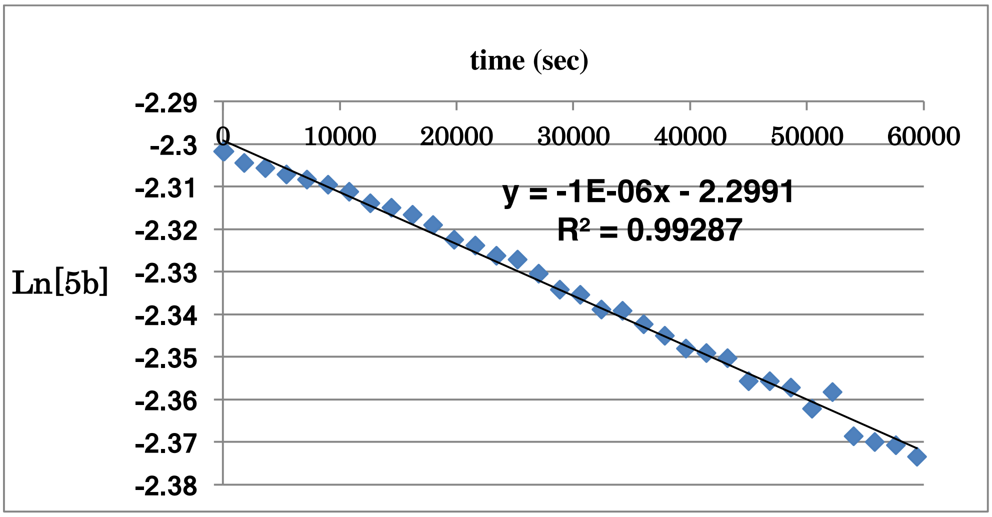

5c (X=NO2)

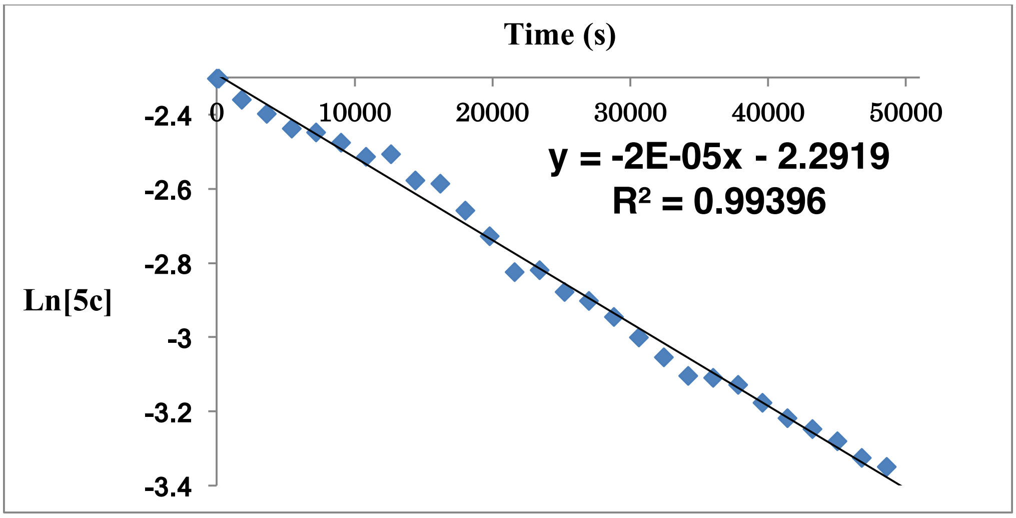

Figure S2. Hydrolysis in methanol-d_4-D_2O (50:50)-NaOD 70°C

2a (X=H)

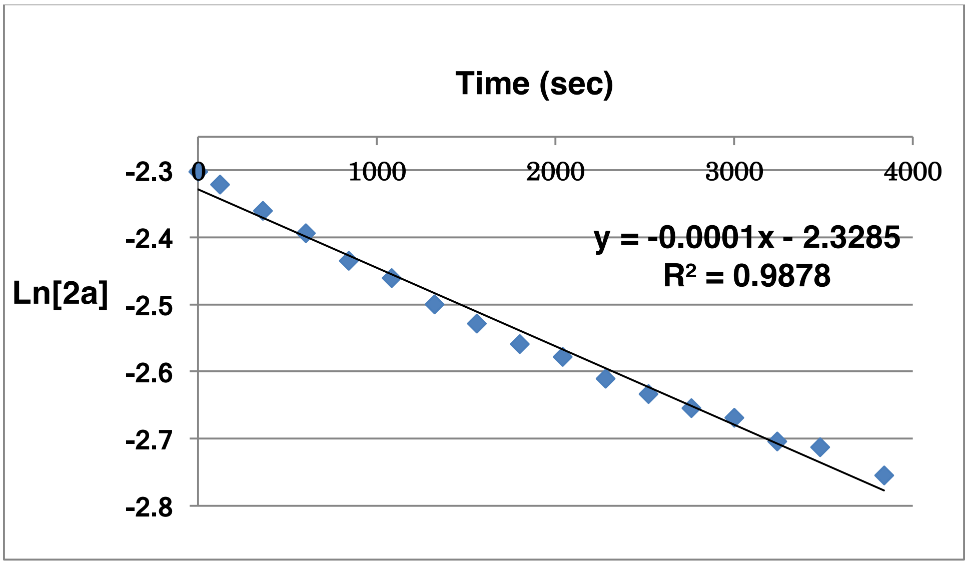

2b (X=Cl)

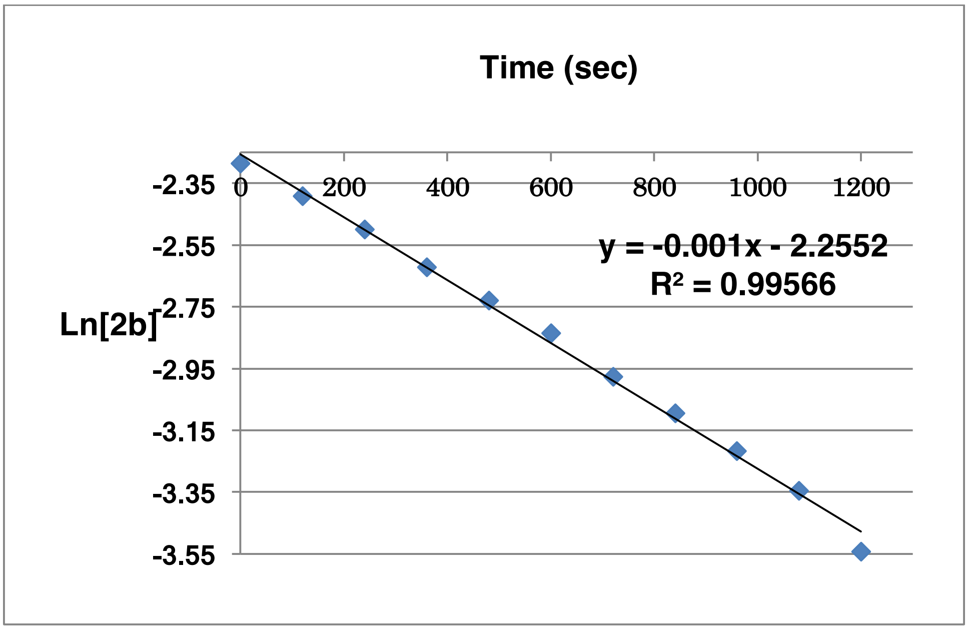

2c (X=NO2)

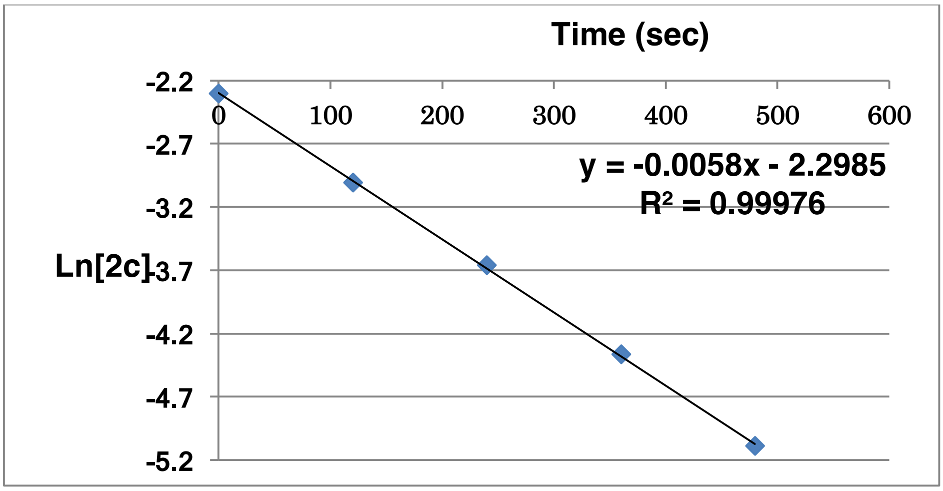

3a (X=H)

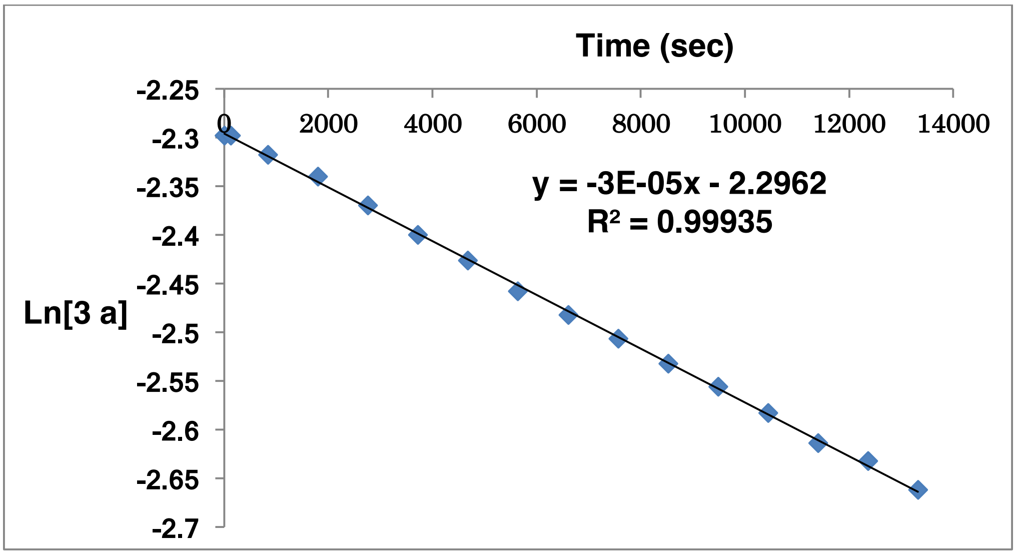

3b(X=Cl)

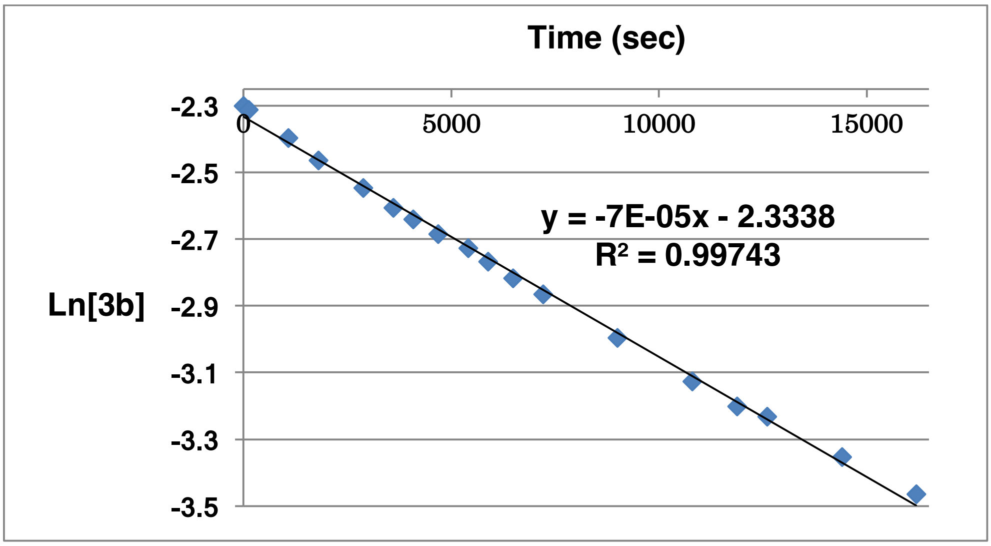

3c (X=NO2)

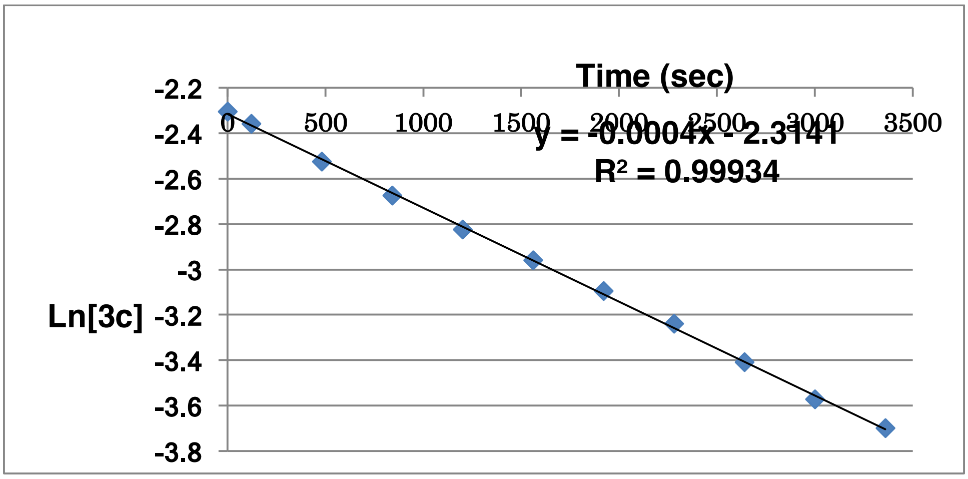

3d(X=Me)

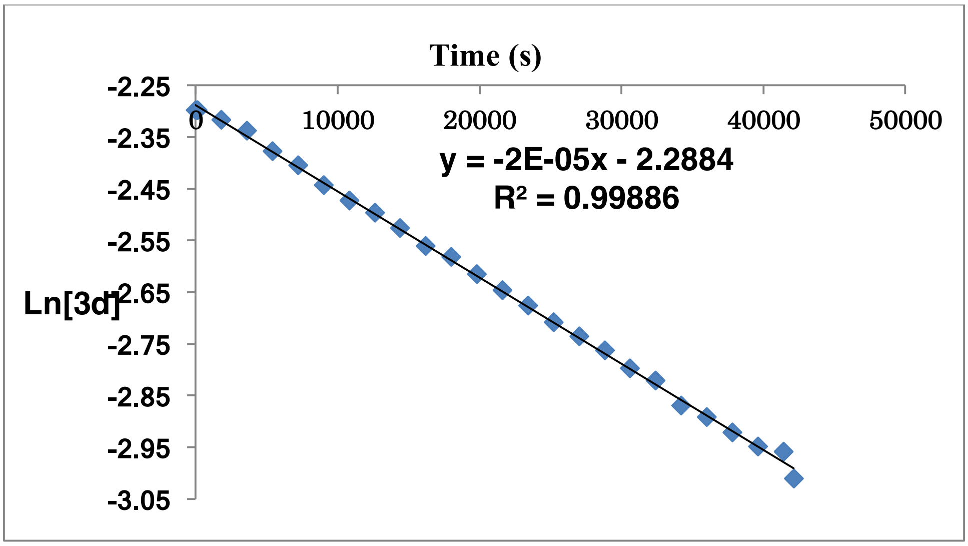

3e (X=MeO)

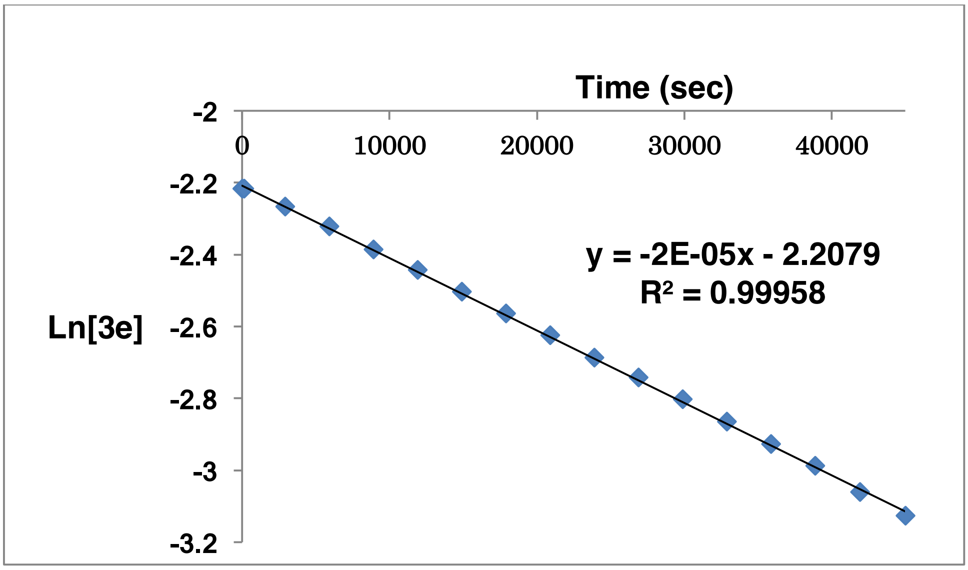

4b (X=Cl)

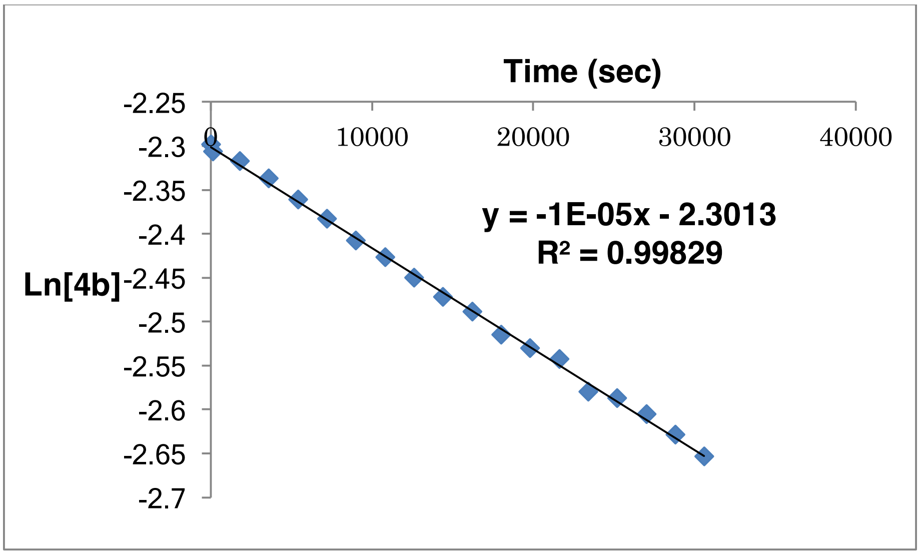

4c (X=NO2)

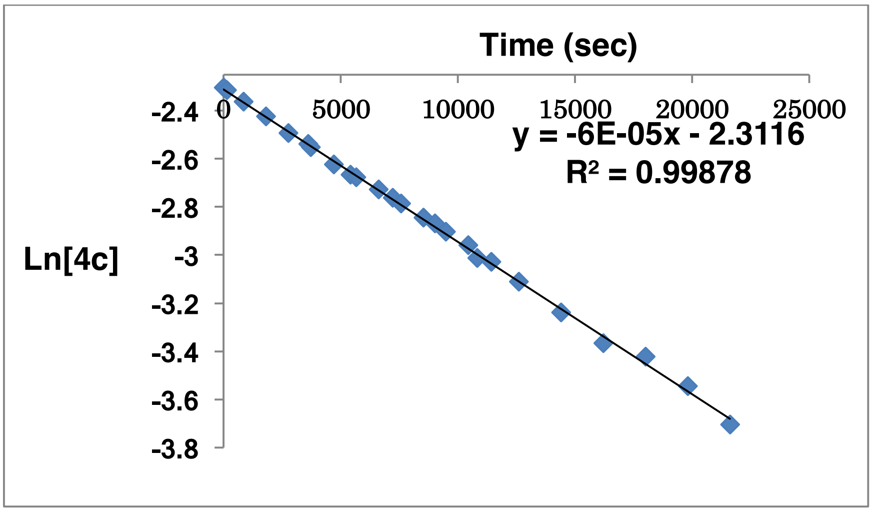

4d (X=Me)

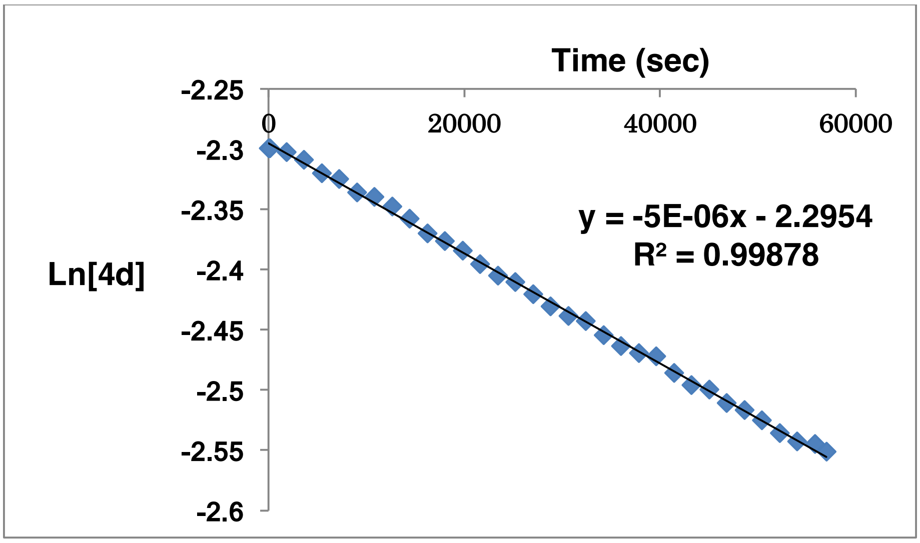

5c (X=NO2)

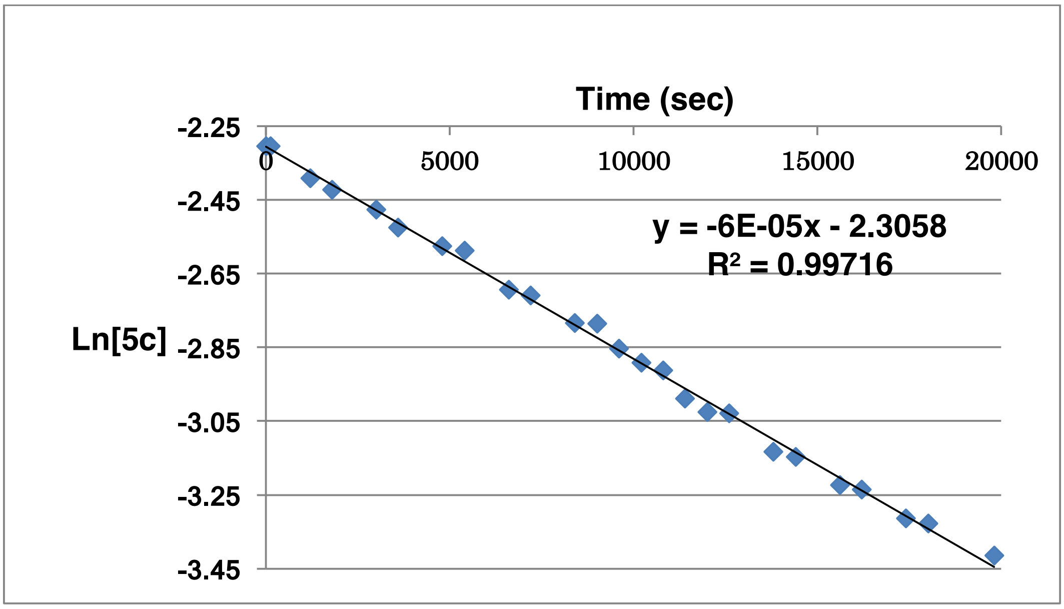
Time (sec)
-2.25
-2.45
-2.65
Ln[5c]
-2.85
-3.05
-3.25
-3.45
0
5000
10000
15000
20000
y = -6E-05x - 2.3058
R² = 0.99716

2. Results of Calculations of Different Model

1. Calculations With Implicit Water Molecules

Table S1. Calculated thermodynamic parameters for the formation of the transition state under implicit solvent conditions at 25 °C.

B3LYP/6-31+G(d), OPT, no solvent model.

Compound	$\Delta G^{\ddagger}_{25\,°C}$ kcal/mol	$\Delta H^{\ddagger}$ kcal/mol	$-T\Delta S^{\ddagger}$ kcal/mol	$\Delta S^{\ddagger}$ cal/(mol•K)	ΔE (ZPE corrected) kcal/mol
**2a**	23.26	12.52	+10.74	-36.04	13.69
**3a**	24.95	14.47	+10.48	-35.15	15.58
**4a**	26.40	15.92	+10.47	-35.12	16.99
**5a**	25.51	13.81	+11.71	-39.26	15.08

2. Model With 4 Explicit Water Molecules

Table S2. Free energy barrier for the formation of the transition state at 25 °C at the B3LYP/6-31+G(d) level considering four water molecules.

B3LYP/6-31+G(d), OPT, IEFPCM (solvent=water)

Compound	$\Delta G^{\ddagger}_{25\,°C}$ kcal/mol	$\Delta H^{\ddagger}$ kcal/mol	$-T\Delta S^{\ddagger}$ kcal/mol	$\Delta S^{\ddagger}$ cal/(mol•K)	ΔE (ZPE corrected) kcal/mol
**2a**	32.03	15.66	+16.37	-54.91	16.90
**3a**	32.47	19.86	+12.61	-42.30	20.00
**4a**	35.09	19.53	+15.56	-52.198	20.59
**5a**	36.19	19.88	+16.31	-54.70	20.99

Table S3. Free energy barrier for the formation of the transition state at 25 °C at the B3LYP/6-311++G(d,p) level considering four water molecules.

B3LYP/6-311++G(d, p), OPT, IEFPCM (solvent=water)

Compound	$\Delta G^{\ddagger}_{25\,°C}$ kcal/mol	$\Delta H^{\ddagger}$ kcal/mol	$-T\Delta S^{\ddagger}$ kcal/mol	$\Delta S^{\ddagger}$ cal/(mol▪K)	ΔE (ZPE corrected) kcal/mol
**2a**	32.04	18.75	+13.29	-44.56	19.00
**3a**	34.06	17.51	+16.549	-55.49	18.75
**4a**	37.45	21.47	+15.989	-53.61	22.53
**5a**	39.36	23.40	+15.969	-53.53	24.44

Table S4. Free energy barrier for the formation of the transition state at 25 °C at the B3LYP/6-31+G(d) level considering four water molecules for *p*-chloro compounds.

B3LYP/6-31+G(d), OPT, IEFPCM (solvent=water)

Compound	$\Delta G^{\ddagger}_{25\,°C}$ kcal/mol	$\Delta H^{\ddagger}$ kcal/mol	$-T\Delta S^{\ddagger}$ kcal/mol	$\Delta S^{\ddagger}$ cal/(mol▪K)	ΔE (ZPE corrected) kcal/mol
**2b**	31.22	14.78	+16.45	-55.16	16.00
**3b**	30.83	14.48	+16.35	-54.86	15.83
**4b**	35.03	18.608	+16.43	-55.09	19.72
**5b**	37.43	20.3189	+17.10	-57.37	21.54

Table S5 Free energy barrier for the formation of the transition state at 25 °C at the B3LYP/6-31+G(d) level considering four water molecules for *p*-nitro compounds.

B3LYP/6-31+G(d), OPT, IEFPCM (solvent=water)

Compound	$\Delta G^{\ddagger}_{25\,°C}$ kcal/mol	$\Delta H^{\ddagger}$ kcal/mol	$-T\Delta S^{\ddagger}$ kcal/mol	$\Delta S^{\ddagger}$ cal/(mol▪K)	ΔE (ZPE corrected) kcal/mol
**2c**	28.75	12.73	+16.02	-53.74	13.86
**3c**	29.19	12.62	+16.57	-55.57	13.96
**4c**	32.35	16.45	+15.90	-53.33	17.50
**5c**	35.03	17.97	+17.064	-57.23	19.19

3. Caluculation Coordinates, Frequecy, thermal values of Transition States

TS: 2a + OH(-) + 5 H2O   M06-2X/6-31+G(d), SMD: sovent=water

NIMG=1 (-328.5862 cm^{-1})

Zero-point correction= 0.329206 (Hartree/Particle)

Thermal correction to Energy= 0.353377

Thermal correction to Enthalpy= 0.354321

Thermal correction to Gibbs Free Energy= 0.276415

Sum of electronic and zero-point Energies= -975.079511

Sum of electronic and thermal Energies= -975.055340

Sum of electronic and thermal Enthalpies= -975.054396

Sum of electronic and thermal Free Energies= -975.132302

Standard orientation:

Center Number	Atomic Number	Atomic Type	Coordinates (Angstroms) X	Y	Z
1	6	0	0.496300	0.165994	0.606737
2	6	0	-1.510976	-2.434479	1.487302
3	6	0	-1.525733	-0.895984	1.559260
4	6	0	-0.127063	-2.308461	0.821949
5	7	0	-0.076696	-0.899254	1.273265
6	8	0	0.131248	1.331041	0.971862
7	1	0	-2.294030	-2.896957	0.884755
8	1	0	-1.468893	-2.902714	2.472706
9	1	0	-0.186302	-2.391832	-0.265375
10	6	0	1.931580	-0.012273	0.157554
11	6	0	2.372998	-1.068046	-0.648781
12	6	0	2.861829	0.925346	0.617028
13	6	0	3.721550	-1.182309	-0.981655
14	1	0	1.665217	-1.787768	-1.043894
15	6	0	4.211552	0.810078	0.284250

16	1	0	2.523478	1.748314	1.239007
17	6	0	4.645931	-0.246017	-0.514764
18	1	0	4.048943	-2.002452	-1.614416
19	1	0	4.921408	1.545942	0.651350
20	1	0	5.696127	-0.338001	-0.777219
21	1	0	-2.090629	-0.429842	0.742202
22	1	0	0.682424	-2.935461	1.204542
23	1	0	-1.805076	-0.434585	2.510617
24	8	0	-0.364503	-0.039590	-1.159572
25	1	0	0.229735	0.414962	-1.778011
26	8	0	-0.028851	3.177339	-1.057412
27	1	0	-0.895559	2.885090	-1.392720
28	8	0	-2.293692	2.360505	1.573562
29	1	0	-1.423985	1.896962	1.478699
30	1	0	0.148601	2.531887	-0.333384
31	1	0	-2.862290	1.737617	2.053129
32	8	0	-2.121351	-1.905171	-1.990701
33	1	0	-1.421620	-1.249006	-1.724222
34	1	0	-2.936682	-1.561581	-1.572521
35	8	0	-4.288272	-0.518092	-0.751808
36	1	0	-3.719592	0.276355	-0.911440
37	1	0	-5.000353	-0.459240	-1.407819
38	8	0	-2.479147	1.510926	-1.126599
39	1	0	-1.661833	0.907003	-1.117399
40	1	0	-2.523482	1.881338	-0.219395

TS: 3a + OH(-) + 5 H_2O M06-2X/6-31+G(d), SMD: sovent=water

NIMG=1 (-352.5835 cm^{-1})

Zero-point correction= 0.358981 (Hartree/Particle)

Thermal correction to Energy= 0.384151

Thermal correction to Enthalpy= 0.385096

Thermal correction to Gibbs Free Energy= 0.304993

Sum of electronic and zero-point Energies= -1014.372067

Sum of electronic and thermal Energies= -1014.346896

Sum of electronic and thermal Enthalpies= -1014.345952

Sum of electronic and thermal Free Energies= -1014.426054

Standard orientation:

Center Number	Atomic Number	Atomic Type	Coordinates (Angstroms) X	Y	Z
1	6	0	-0.518209	0.164716	-0.520763
2	6	0	0.904359	-3.179562	-1.030380
3	6	0	2.133491	-2.266438	-1.105798
4	6	0	1.549336	-0.857363	-1.293929
5	6	0	-0.116598	-2.295223	-0.325120
6	7	0	0.120623	-0.985167	-0.940606
7	8	0	-0.207549	1.257269	-1.117280
8	1	0	0.546632	-3.430963	-2.035316
9	1	0	2.685996	-2.315694	-0.161728
10	1	0	2.819256	-2.537799	-1.912310
11	1	0	0.083991	-2.267379	0.755141
12	1	0	1.093609	-4.109073	-0.487374
13	6	0	-1.971950	0.017143	-0.099468
14	6	0	-2.401149	-0.699985	1.023157
15	6	0	-2.927049	0.639447	-0.909305
16	6	0	-3.758741	-0.791622	1.324980
17	1	0	-1.674499	-1.175025	1.673567
18	6	0	-4.286651	0.543641	-0.610781
19	1	0	-2.601411	1.203454	-1.778326
20	6	0	-4.706688	-0.171871	0.509065
21	1	0	-4.074960	-1.345825	2.204337
22	1	0	-5.014923	1.031354	-1.252799
23	1	0	-5.764076	-0.244293	0.747838
24	1	0	2.038482	-0.127456	-0.637163

25	1	0	-1.148499	-2.618118	-0.482355
26	1	0	1.649675	-0.500984	-2.324904
27	8	0	0.274747	0.376631	1.220635
28	1	0	-0.352509	0.901187	1.744086
29	8	0	-0.217771	3.470739	0.490386
30	1	0	0.643039	3.325715	0.921378
31	8	0	2.131368	2.381777	-1.847225
32	1	0	1.300044	1.868777	-1.679055
33	1	0	-0.312812	2.669026	-0.079911
34	1	0	2.755170	1.736931	-2.216760
35	8	0	2.051651	-1.280600	2.368599
36	1	0	1.335383	-0.704636	1.986135
37	1	0	2.861894	-0.980231	1.908575
38	8	0	4.197303	-0.022420	0.960895
39	1	0	3.597861	0.765192	0.970336
40	1	0	4.893591	0.179617	1.605205
41	8	0	2.330525	1.988883	0.961342
42	1	0	1.536034	1.362971	1.039061
43	1	0	2.375584	2.213890	0.007814

TS: 4a + OH(-) + 5 H_2O M06-2X/6-31+G(d), SMD: sovent=water

NIMG=1 (-347.8497 cm^{-1})

Zero-point correction= 0.394269 (Hartree/Particle)

Thermal correction to Energy= 0.420704

Thermal correction to Enthalpy= 0.421649

Thermal correction to Gibbs Free Energy= 0.337737

Sum of electronic and zero-point Energies= -1091.713231

Sum of electronic and thermal Energies= -1091.686795

Sum of electronic and thermal Enthalpies= -1091.685851

Sum of electronic and thermal Free Energies= -1091.769763

Standard orientation:

Center Number	Atomic Number	Atomic Type	Coordinates (Angstroms) X	Y	Z
1	6	0	0.543490	0.016956	0.632701
2	6	0	1.221968	3.521289	-0.111601
3	6	0	0.000976	3.584311	0.852491
4	6	0	-0.663687	2.217539	0.614095
5	6	0	-1.319579	2.244801	-0.787618
6	6	0	-0.093146	2.157539	-1.737073
7	6	0	1.081691	2.119566	-0.739232
8	7	0	0.534308	1.377131	0.414191
9	8	0	-0.012436	-0.417737	1.702197
10	1	0	1.201751	4.300807	-0.878365
11	1	0	0.324035	3.681625	1.892793
12	1	0	-0.681685	4.407437	0.625078
13	1	0	-1.894586	3.165849	-0.924612
14	1	0	-1.991742	1.397195	-0.928075
15	1	0	-0.123669	1.248701	-2.343544
16	1	0	-0.011101	3.017715	-2.408089
17	1	0	2.013437	1.705535	-1.120839
18	1	0	2.163979	3.605265	0.437534
19	6	0	1.802205	-0.703089	0.176736
20	6	0	2.631452	-1.218953	1.176702
21	6	0	2.179343	-0.851448	-1.162431
22	6	0	3.821751	-1.869192	0.847977
23	1	0	2.340912	-1.109583	2.217667
24	6	0	3.365897	-1.503997	-1.491463
25	1	0	1.535812	-0.469260	-1.948852
26	6	0	4.191817	-2.013926	-0.487791
27	1	0	4.456514	-2.263406	1.636667
28	1	0	3.643202	-1.619095	-2.535640
29	1	0	5.114809	-2.524802	-0.747405
30	1	0	-1.303502	1.862662	1.421862

31	8	0	-0.663616	-0.687685	-0.743133
32	1	0	-0.476122	-1.639376	-0.684281
33	8	0	-1.252910	-2.859845	1.467774
34	1	0	-0.741213	-2.018802	1.550193
35	1	0	-2.056638	-2.583016	0.995026
36	8	0	-2.297525	-0.038275	3.083333
37	1	0	-2.531811	0.894085	3.212272
38	1	0	-1.422901	-0.027417	2.620717
39	8	0	-2.303037	-0.747902	-2.878030
40	1	0	-1.642968	-0.639251	-2.140168
41	1	0	-2.938867	-1.392595	-2.507720
42	8	0	-3.029237	-0.615416	0.388409
43	8	0	-3.906647	-2.562645	-1.308896
44	1	0	-3.712096	-1.930758	-0.577474
45	1	0	-4.846398	-2.437004	-1.512226
46	1	0	-2.090945	-0.586448	0.004626
47	1	0	-2.930159	-0.458308	1.349315

TS: 5a + OH(-) + 5 H_2O M06-2X/6-31+G(d), SMD: sovent=water

NIMG=1 (-354.0983 cm^{-1})

Zero-point correction= 0.457881 (Hartree/Particle)

Thermal correction to Energy= 0.487547

Thermal correction to Enthalpy= 0.488491

Thermal correction to Gibbs Free Energy= 0.399682

Sum of electronic and zero-point Energies= -1245.424852

Sum of electronic and thermal Energies= -1245.395187

Sum of electronic and thermal Enthalpies= -1245.394243

Sum of electronic and thermal Free Energies= -1245.483051

Standard orientation:

Center	Atomic	Atomic	Coordinates (Angstroms)		
Number	Number	Type	X	Y	Z

1	6	0	-0.714864	-0.093595	-0.391890
2	6	0	-0.066436	3.491962	-0.585342
3	6	0	1.311039	2.887819	-0.967042
4	6	0	1.275153	1.513911	-0.269408
5	6	0	1.399283	1.773377	1.254736
6	6	0	0.012882	2.349373	1.631354
7	6	0	-0.696950	2.372349	0.264017
8	7	0	-0.167926	1.180718	-0.433136
9	8	0	-0.183108	-0.997495	-1.124926
10	1	0	0.016580	4.421401	-0.015397
11	1	0	1.405845	2.764641	-2.049257
12	1	0	2.158095	3.482133	-0.611621
13	1	0	2.222516	2.465735	1.457158
14	1	0	1.599702	0.838777	1.783695
15	1	0	-0.506907	1.698442	2.339817
16	1	0	0.071018	3.352885	2.063025
17	1	0	-1.784144	2.403974	0.298340
18	1	0	-0.672095	3.687705	-1.474463
19	6	0	-2.237342	-0.125327	-0.294872
20	6	0	-2.930110	-0.551834	-1.431595
21	6	0	-2.964468	0.244808	0.841974
22	6	0	-4.324575	-0.603560	-1.438410
23	1	0	-2.371087	-0.846640	-2.315106
24	6	0	-4.356872	0.188370	0.838668
25	1	0	-2.439592	0.568157	1.735863
26	6	0	-5.042161	-0.234716	-0.301817
27	1	0	-4.847591	-0.934586	-2.331386
28	1	0	-4.908033	0.471657	1.731009
29	1	0	-6.127668	-0.278796	-0.301597
30	8	0	-0.318196	-0.745201	1.378534
31	1	0	-1.041158	-1.374604	1.539957
32	8	0	-1.021644	-3.456130	-0.294989

33	1	0	-0.792329	-2.556348	-0.640555
34	1	0	-0.307002	-3.627024	0.342746
35	8	0	1.807122	-2.701268	-1.764853
36	1	0	2.634854	-2.408810	-2.175787
37	1	0	1.247869	-1.895040	-1.687540
38	8	0	0.920001	-0.605489	3.779477
39	1	0	0.420508	-0.601695	2.918483
40	1	0	1.846532	-0.739051	3.498012
41	8	0	1.523320	-2.625486	1.072644
42	8	0	3.413532	-1.139141	2.387862
43	1	0	2.810773	-1.692740	1.832195
44	1	0	3.526312	-0.315782	1.886617
45	1	0	0.842300	-1.881181	1.115794
46	1	0	1.762631	-2.702418	0.125870
47	6	0	2.327659	0.536162	-0.734236
48	1	0	2.215326	-0.432944	-0.227456
49	1	0	3.297605	0.963073	-0.434010
50	8	0	2.312563	0.352265	-2.140272
51	6	0	3.590559	0.002988	-2.644131
52	1	0	4.023138	-0.840423	-2.087760
53	1	0	3.465017	-0.278173	-3.691834
54	1	0	4.280974	0.853682	-2.572711

----

4. Spectra data of new compounds

Figure S3-1 5a ^{1}H-NMR

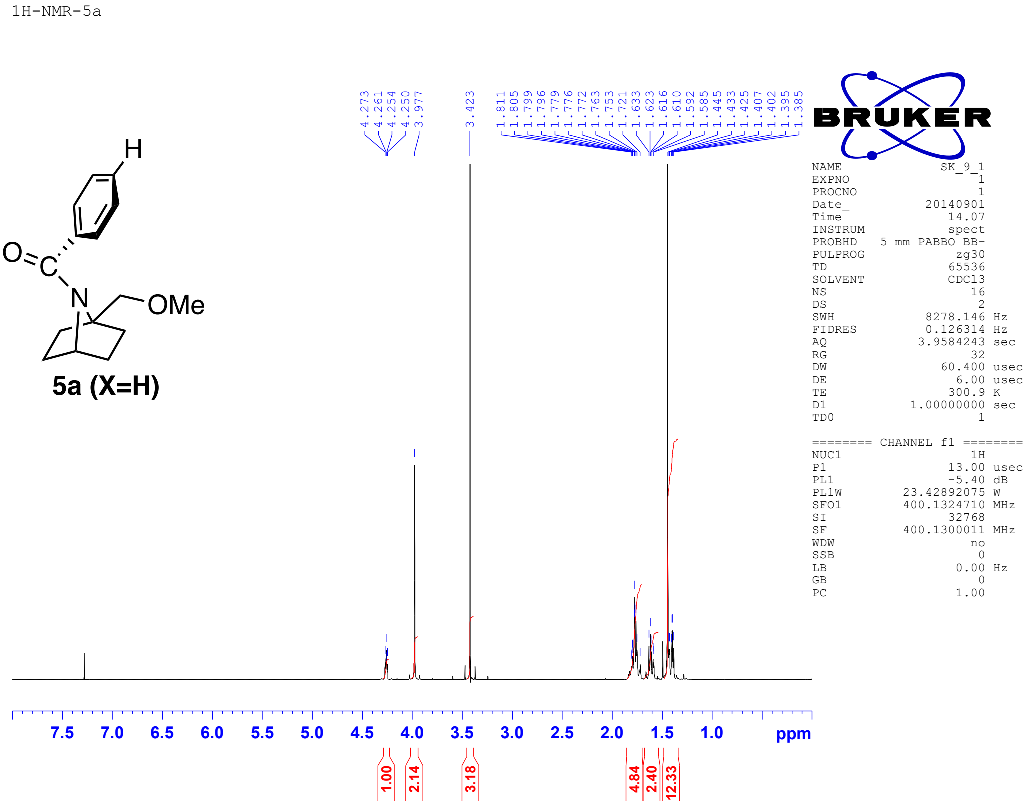

Figure S3-2 5a ^{13}C-NMR

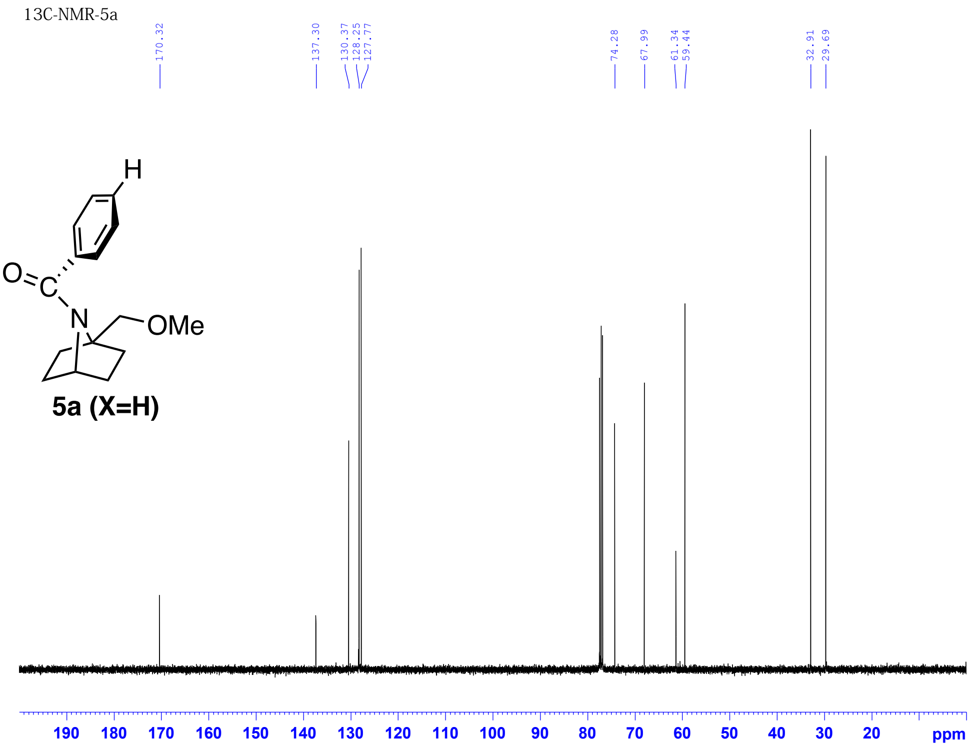

Figure S4-1 5b ^{1}H-NMR

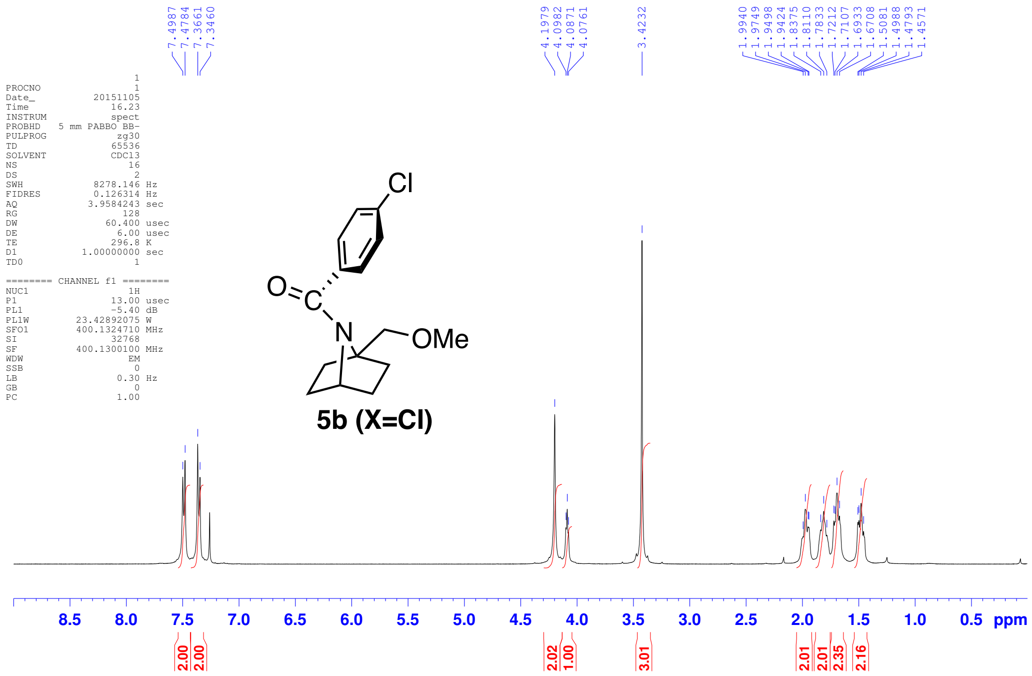

Figure S4-2 5b ^{13}C-NMR

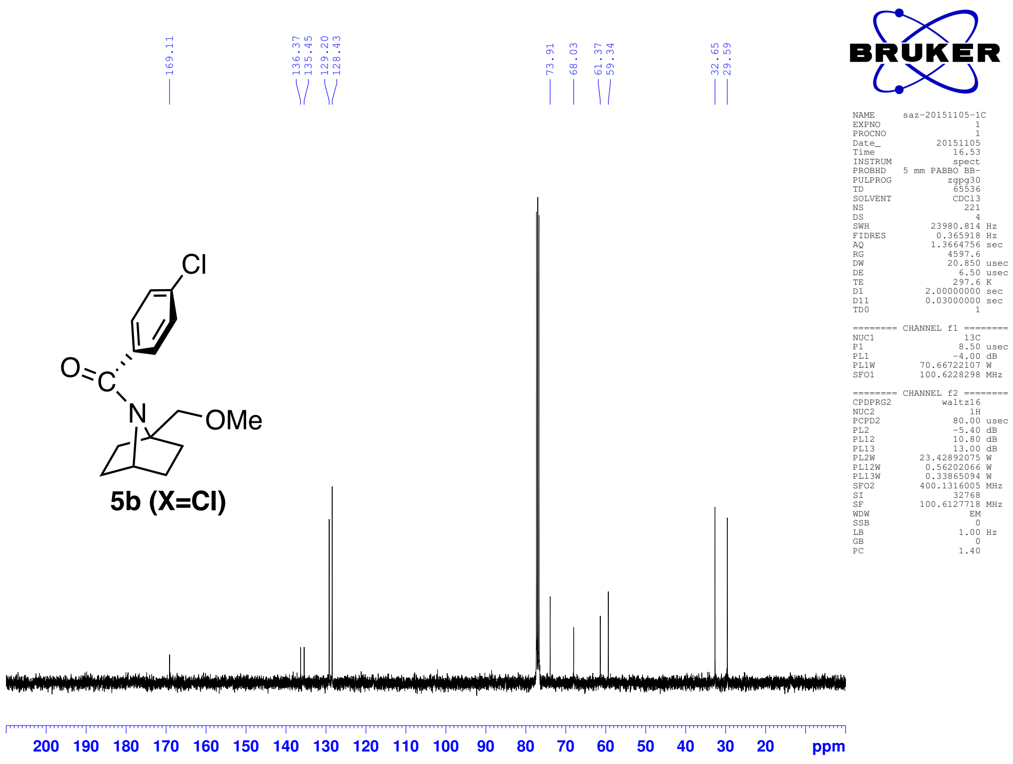

Figure S5-1 5c ^{1}H-NMR

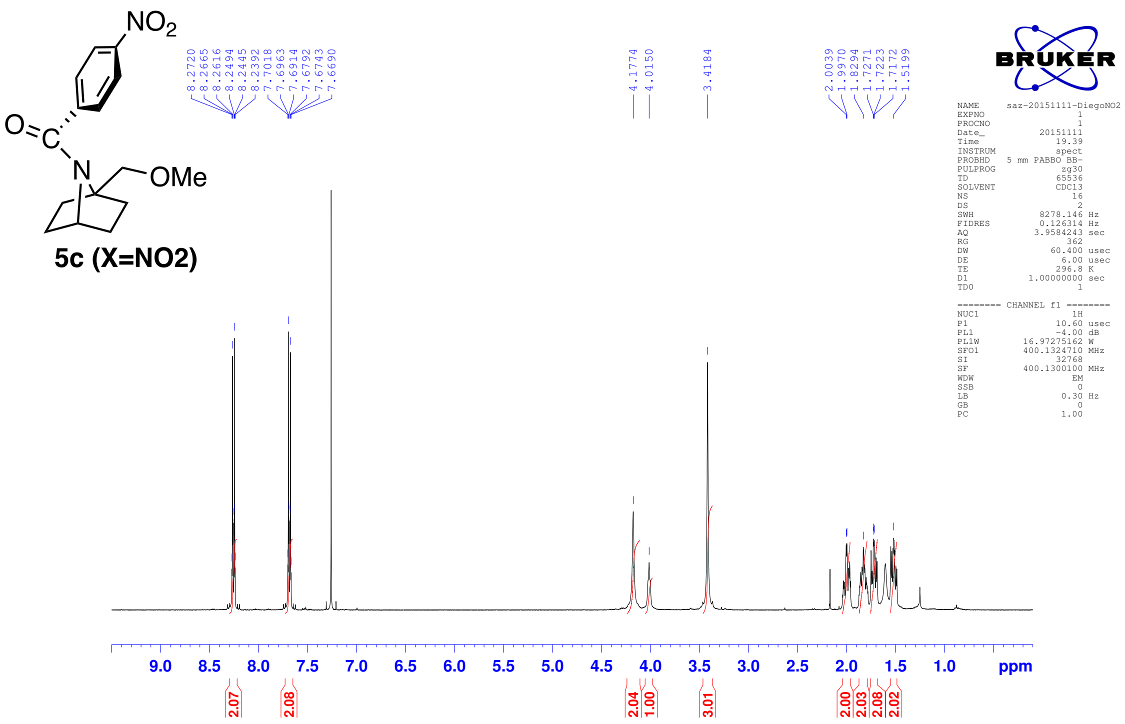

Figure S5-1 5c ^{13}C-NMR

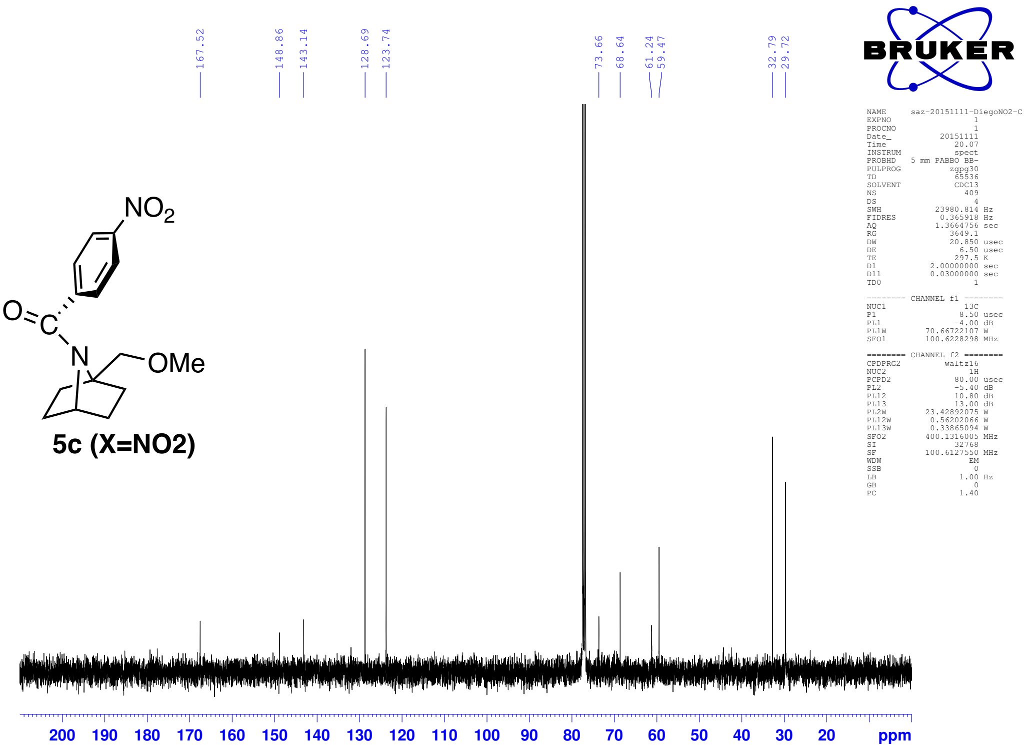

Supplement: Supplementary file 1 [file molecules-23-02363-s001.pdf]
